# Ferritin light chain promotes the reprogramming of glioma immune microenvironment and facilitates glioma progression

**DOI:** 10.7150/thno.82975

**Published:** 2023-06-26

**Authors:** Hongjiang Li, Chao Yang, Yanfei Wei, Xueyuan Li, Wei Jiang, Yiran Xu, Lifeng Li, Rongqun Guo, Di Chen, Peng Gao, Haohao Zhang, Hui Qin, Zhenyu Zhang, Xianzhi Liu, Dongming Yan

**Affiliations:** 1 Department of Neurosurgery, The First Affiliated Hospital of Zhengzhou University, Zhengzhou, 450052, China.; 2 The Application Center for Precision Medicine, Academy of Medical Science, Zhengzhou, 450052, China.; 3 Henan Key Laboratory of Child Brain Injury and Henan Clinical Research Center for Child Neurological Disorders, Institute of Neuroscience and The Third Affiliated Hospital of Zhengzhou University, Zhengzhou, 450052, China.; 4 Cancer Center, The First Affiliated Hospital of Zhengzhou University, Zhengzhou, 450052, China.; 5 National Engineering Laboratory for Internet Medical Systems and Applications, The First Affiliated Hospital of Zhengzhou University, Zhengzhou, 450052, China.; 6 Department of Hematology, The First Affiliated Hospital of Zhengzhou University, Zhengzhou, 450052, China.; 7 Department of Endocrinology, The First Affiliated Hospital of Zhengzhou University, Zhengzhou, 450052, China.; 8 Department of Pathology, The First Affiliated Hospital of Zhengzhou University, Zhengzhou, 450052, China.

**Keywords:** Glioblastoma, Ferritin light chain, Tumor-associated macrophages, macrophage polarization, PD1

## Abstract

**Background:** Tumor-associated macrophages (TAMs), the most abundant non-tumor cell population in the glioma microenvironment, play a crucial role in immune evasion and immunotherapy resistance of glioblastoma (GBM). However, the regulatory mechanism of the immunosuppressive TME of GBM remains unclear.

**Methods:** Bioinformatics were used to analyse the potential role of ferritin light chain (FTL) in GBM immunology and explore the effects of FTL on the reprogramming of the GBM immune microenvironment and GBM progression.

**Results:** The FTL gene was found to be upregulated in TAMs of GBM at both the bulk and single-cell RNA-seq levels. FTL contributed to the protumor microenvironment by promoting M2 polarization in TAMs via inhibiting the expression of iPLA_2_β to facilitate the ferroptosis pathway. Inhibition of FTL in TAMs attenuated glioma angiogenesis, promoted the recruitment of T cells and sensitized glioma to anti-PD1 therapy.

**Conclusion:** Our study suggested that FTL promoted the development of an immunosuppressive TME by inducing M2 polarization in TAMs, and inhibition of FTL in TAMs reprogrammed the TME and sensitized glioma to anti-PD1 therapy, providing a new strategy for improving the therapeutic effect of anti-PD1.

## Introduction

Glioblastoma (GBM) is the most common primary malignant brain tumor in adults, characterized by its aggressive growth and invasive nature within the central nervous system^1^. Although accepted aggressive treatments, GBM patients have poor outcomes, with a median survival time of approximately 15 months^2^. The resistance of GBM to multiple treatments is due to their unique immunosuppressive tumor microenvironment (TME). Therefore, it is urgent to explore the regulatory mechanisms of the immunosuppressive TME of GBM to develop more effective therapies.

Macrophages, primary immune cells that reside in and around the glioma TME, mainly polarize into classically activated (M1) macrophages and alternatively activated (M2) macrophages^3^. Tumor-associated macrophages (TAMs), abundant infiltrating immune cells in the glioma TME, resembles M2 macrophages by interacting with tumor cells and are associated with tumor progression and tumor-specific immunosuppression^4^. Hence, the exploration of the key molecular mechanisms that regulate TAMs is expected to identify new potential therapeutic targets, ultimately to improve therapeutic efficacy of GBM.

Ferroptosis is a newly established form of iron-dependent cell death, characterized by the accumulation of lipid peroxides and reactive oxygen species (ROS), and plays an important role in the occurrence and therapy of various diseases^5^. In the last decade, progress has been made in exploring the role of ferroptosis in cancer therapy^6^. However, the mechanism of ferroptosis in the TME of GBM has not been well investigated.

In the present study, the differential expression of ferroptosis-related genes was analyzed based on the public databases, and ferritin light chain (FTL) was identified to undergo significant changes in GBM. Moreover, the expression of FTL was analyzed in bulk and at single-cell RNA-seq level, and a comprehensive analysis of the correlation between FTL expression and glioma-infiltrating immune cells was performed, aiming to reveal the potential role of FTL in glioma immunology. We further demonstrated that FTL facilitated the protumor microenvironment by promoting M2 polarization in TAMs. Finally, inhibition of FTL in TAMs attenuated glioma angiogenesis and sensitized glioma to anti-PD1 therapy.

## Materials and methods

### Ethical statements

All procedures involving mice were approved by and under the requirements of the Animal Care and Use Committee of the First Affiliated Hospital of Zhengzhou University. Studies with human tissue were conducted in accordance with the Declaration of Helsinki and all patients gave written informed consent to participate in the study. The study received the approval of the Ethics Committee of the First Affiliated Hospital of Zhengzhou University (approval number: 2019-KY-176).

### Animals

C57BL/6N mice were obtained from GemPharmatech Co. Ltd., Nanjing, China. All mice were maintained in a specific pathogen-free facility and kept in a 12 h light/12 h dark cycle with 3-4 animals per cage, sustained by free water and a standard rodent diet.

### Patient samples

Patients underwent surgical treatment in the Department of Neurosurgery, the First Affiliated Hospital of Zhengzhou University. Specimens were histologically diagnosed as GBM according to the WHO classification. Human para-tumor tissue (PT) of GBM was used as a control.

### Cell culture

The murine glioma cell line GL261, monocyte-macrophage cell line RAW264.7, and human monocyte cell line THP-1 were purchased from the Chinese Academy of Sciences Cell Bank and Procell Life Science and Technology Co., Ltd, China. Patient-derived glioma cell line GBM-Z1 was established from resected human glioma tissues histologically diagnosed as GBM obtained from a 59-year-old man. RAW264.7 and GL261 cells were cultured in Dulbecco's modified Eagle's medium (DMEM, Thermo Fisher Scientific, USA) supplemented with 10% Fetal Bovine Serum (FBS, Thermo Fisher Scientific, USA). GBM-Z1 glioma cells were cultured in DMEM/F12 (Thermo Fisher Scientific, USA) supplemented with 10% FBS. THP-1 cells were cultured in RPMI-1640 (Thermo Fisher Scientific, USA) supplemented with 10% FBS and induced differentiation into macrophage by 100 ng/ml Phorbol 12-myristate 13-acetate (Sigma-Aldrich, USA).

### Bioinformatics analysis

To explore the expression and prognostic role of FTL in GBM, Gliovis (http://gliovis.bioinfo.cnio.es/), Chinese Glioma Genome Atlas (CGGA) and GEO databases (http://www.ncbi.nlm.nih.gov/geo) were selected. Normalized RSEM gene-level RNA-seq and corresponding clinical data of Rembrandt were obtained from Gliovis. The expression matrix and clinical data of GSE4290 were downloaded from the GEO database. The mRNA expression (CGGA325, CGGA693) and clinical data were obtained from CGGA. Using the R package “survival”, glioma patients were divided into high- and low-risk groups based on the median risk score^7^. The receiver operating characteristics (ROC) curves (3-year, 5-year, and 10-year) were analyzed to explore the prognostic value of the FTL-based classifier by using the R package “timeROC”^8^. For clinical feature analysis, RNA sequencing data were used for subtype analysis of the histology, grade, IDH status, 1p/19q status, gender, age, and progression status.

### Immunofluorescence staining

Animals were sacrificed by isoflurane inhalation and were transcardially perfused with 0.9% saline followed by 4% paraformaldehyde (PFA, Solarbio, China). Brain tissue specimens were embedded in paraffin, and 20 μm coronal sections were created using a pathological microtome (RM2016, Leica, Germany). Brain sections were deparaffinized, rehydrated and immersed in EDTA antigen retrieval buffer (pH 8.0) for antigen retrieval. Sections were treated with 3% hydrogen peroxide to block endogenous peroxidase and followed by the first primary antibody incubation in 3% horse serum or fetal bovine serum overnight at 4 °C. Sections were incubated with the corresponding secondary antibody marked with HRP for 1 h and then incubated with CY3-TSA solution for 10 min. Sections were subjected to microwave treatment by immersing in EDTA antigen retrieval buffer (pH 8.0) and maintained at a sub-boiling temperature for 8 min, standing for 8 min and then again maintained at a sub-boiling temperature for 7 min to remove the primary antibodies and secondary antibodies. Sections were incubated with the second primary antibody and corresponding secondary antibody marked with HRP and incubated with the FITC-TSA solution for 10 min, and then subjected to microwave treatment. Sections were incubated with the third primary antibody and corresponding secondary antibody marked with CY5 for 1 h and incubated with a 4′,6′-diamidino-2-phenylindole (DAPI, Solarbio, China) solution for 10 min. Fluorescence images were acquired using a fluorescence microscope (Olympus, Tokyo, Japan).

The primary antibodies were used: anti-FTL (1:200, ab109373, Abcam), anti-iPLA_2_β (1:200, ab244247, Abcam), anti-CD163 (1:200, ab244247, Abcam), anti-CD3 (1:200, ab16669, Abcam), anti-CD31 (1:200, ab222783, Abcam) and anti-CD8 (1:200, ab217344, Abcam).

### Immunohistochemistry and HE staining

For immunohistochemical (IHC) staining, brain tissues were fixed and paraffin-embedded with 5 μm sections. After deparaffinization, rehydration, antigen retrieval, quenching of endogenous peroxidase and blocking, sections were incubated with primary antibodies overnight at 4°C. The sections were then incubated with the secondary antibody conjugated by horseradish peroxidase (HRP) for 60 min and developed with a DAB chromogenic solution. For HE, the whole brain tissues of the mice were harvested at 3 weeks, fixed by 4% PFA, embedded in paraffin, and cut into 15-μm coronal sections. The slide with the largest tumor area was stained with hematoxylin and eosin and visualized using a light microscope.

The primary antibodies were used: anti-FTL (1:100, ab109373, Abcam), anti-SOX2 (1:200, MA1-014, Thermo Fisher), anti-IBA1 (1:200, 10904-1-AP, Proteintech), anti-CD11B (1:200, ab133357, Abcam), anti-ki67 (1:200, ab15580, Abcam), anti-iPLA_2_β (1:200, ab244247, Abcam), anti-IL-1β (1:200, ab254360, Abcam), and anti-CD163 (1:200, ab182422, Abcam).

### Gene knockdown and overexpression

The overexpression and knockdown of FTL and overexpression of iPLA_2_β lentiviruses were constructed by Genechem Co., Ltd. (Shanghai, China) and Obio Technology Corp., Ltd. (Shanghai, China), and transfected into THP-1-induced macrophages and RAW264.7 macrophages, which are routinely used to study macrophage function, including macrophage polarization.

### ELISA

An ELISA kit (R&D Systems) was used to measure the secreted IL-10 and TNF-α proteins in the cell culture supernatant of conditioned macrophages for 48 h according to the manufacturer's instructions.

### Iron and ROS assay

The relative iron concentration in macrophages was measured with the Iron Assay Kit (ab83366, Abcam). Briefly, cells were rapidly homogenized in an iron assay buffer and centrifuged at 16,000×g for 10 min at 4 ℃. The supernatant was collected and then iron assay buffer was added respectively to measure ferrous iron. Iron probes were added and incubated at 37 ℃ for 1 h. Optical density was measured at 593 nm, and a standard curve line was used for the iron concentration calculation.

The level of intracellular ROS was determined using a ROS assay kit (Abcam) according to the manufacturer's instructions. In brief, cells were washed and incubated with 2,7-dichlorofluorescein diacetate (DCFDA, Thermo Fisher Scientific, USA) at 37 °C for 30 min. The production of ROS was measured using flow cytometry.

### Western blotting and immunoprecipitations

Western blot was performed and analyzed according to the manufacturer's manual. The samples were lysed in a RIPA buffer with a protease inhibitor mixture for 30 min. After centrifugation at 12000×g for 10 min at 4 °C, the supernatant was separated by SDS-polyacrylamide gel electrophoresis (PAGE). The separated proteins were electro-blotted and then incubated overnight at 4 °C with rabbit anti-FTL (1:1000, ab109373, Abcam), rabbit anti-iPLA_2_β (1:1000, 22030-1-AP, Proteintech), and mouse anti-GAPDH (1:1000, ab8245, Abcam). Secondary antibodies conjugated to horseradish peroxidase and an Ultrasensitive Enhanced Chemiluminescence Detection kit were used as the visualization reagent. Immunoreactive bands were measured by ImageJ software.

For the immunoprecipitation experiments, the procedure was performed as previous study^9^. Briefly, cells were homogenized in a co-immunoprecipitation (CO-IP) buffer containing 50 mM Tris-HCl, pH 7.5, 200 mM NaCl, 5 mM MgCl_2_, 1% NP-40, 10% glycerol, 1 mM DTT, 1 mM PMSF, 50 mM NaF, 1 mM Na_3_VO_4_, and protease inhibitors. The lysates were centrifugated and incubated with antibodies for 2 h and Protein G beads overnight at 4 °C. Beads were subjected to SDS-PAGE and then transferred to nitrocellulose membranes. The membranes were immunoblotted with indicated antibodies based on the manufacturer's recommendations. Analysis of the data was performed using ImageJ software.

### Quantitative reverse transcription polymerase reaction

According to the manufacturer's protocol, total RNA was extracted from samples based on the TRI reagent (Sigma). cDNA was synthesized based on the GoScript™ reverse transcription system. GAPDH was used as an internal control. Analysis of data was performed according to the 2^-ΔΔCT^ method. All primer sequences are listed in **Table S1**.

### Colony formation assay

THP-1-induced macrophages were transfected with LV-NC, LV-FTL and LV-shFTL. GBM-Z1 cells were cultured in the culture supernatant of conditioned macrophage. Cells were fixed with 4% PFA and stained with 0.1% crystal violet at 2 weeks. The images of colonies were captured using a digital camera.

### Transwell migration/invasion assays

The transwell chambers (Corning, USA) paved with and without a matrigel mix (Corning, USA) were used to assess the migration and invasion ability. GBM-Z1 cells were inoculated to the upper chamber and conditioned macrophages were inoculated to the bottom chamber. After incubation for 48 h, the cells on the upper surface of the membrane were removed. Cells traversing the membrane were fixed and then stained by crystal violet, and images were taken by an inverted microscope.

### Intracranial glioma model

The animals were anesthetized using isoflurane, and an analgesic was injected before surgery. Using a stereotactic frame, cells were implanted into the right corpus striatum of 4 weeks old C57BL/6N mice at a speed of 0.25 μL/min by a micro-infusion syringe pump. The animals were sacrificed at 3 weeks post-tumor implantation. Bioluminescence imaging was used to assess tumor growth.

### Isolation of tumor-infiltrating immune cells

Three weeks after tumor implantation, mice were anesthetized and perfused transcardially with PBS. The brain was mechanically isolated in cold PBS using a Wheaton Potter-Elvehjem tissue grinder, and then dissociated enzymatically. The enzymatic reaction was stopped by adding Hank's balanced salt solution with calcium and magnesium (Thermo Fisher Scientific, USA). A single-cell suspension was obtained through a 70μm cell strainer and then mixed with a 30% Percoll gradient to remove the myelin, and overlayered with 5ml PBS. After centrifugation at 950×g for 20min without acceleration, the cells were collected.

### Flow cytometry

Antibodies for flow cytometry were purchased from Biolegend and used according to the manufacturer's recommendations. Cells were performed on a BD FACSCelesta flow cytometer (BD Biosciences) and analyzed using the FlowJo software V10. In brief, cells were selected by FSC-A and SSC-A based on size. Then, doublets were removed using FSC-A versus FSC-H. Anti-CD163-BV421 (Biolegend, USA) and anti-CD11b-FITC (Biolegend, USA) were used to detect CD11b^+^ CD163^+^ macrophages.

### Animal Experiments

Mice with a similar weight and age were randomized into three experimental groups. For co-implantation, luciferase labeled GL261 cells (4×10^5^ cells per mouse) mixed with 1×10^5^ conditioned RAW264.7 macrophages were implanted into the right corpus striatum of C57BL/6N mice. Bioluminescence imaging was used to measure the tumor 3 weeks after implantation. For anti-PD-1 therapy, 10 mg/kg of anti-mouse PD-1 Ab was administered intravenously (via tail vein injection) to the mice 5 times every 3 days, starting on day 5 after tumor injection.

### Statistical analysis

Statistical analysis was performed using GraphPad Prism 6 and the statistical tests used are indicated in the individual figure legends. Each experiment was carried out at least three times, and all results are presented as the mean ± SD. The χ^2^-test, Student's t-test, and one-way ANOVA test were performed to analyze statistical significance. P<0.05 was considered statistically significant.

## Results

### FTL is highly expressed in TAMs and correlated with prognosis in GBM

**Figure 1:**
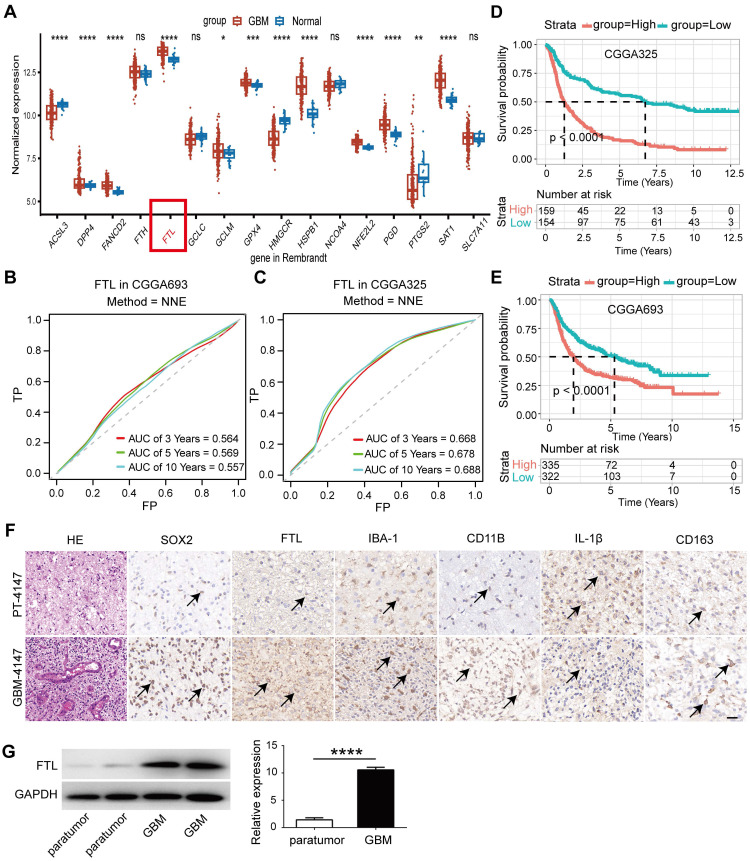
**FTL was identified as a prognostic**-**related gene in glioma. (A)** The expression level of ferroptosis-related genes was compared between normal and GBM tissue based on the Rembrandt database. **(B, C)** AUC of time-dependent ROC curves verified the prognostic performance of the risk score based on FTL in the CGGA693 and CGGA325 databases. **(D, E)** The correlation of FTL expression level with survivals based on the CGGA325 and CGGA693 databases. **(F)** HE and IHC staining of expression levels of FTL and tumor marker SOX2, microglial marker IBA-1, macrophage marker CD11B, M1 macrophage phenotype marker IL-1β, and M2 macrophage phenotype marker CD163 in GBM-4408 and PT adjacent to GBM (scale bars, 50 μm). Representative positives are indicated by an arrow. **(G)** The protein expression level of FTL in GBM and PT adjacent to GBM.

Ferroptosis is a novel form of iron-dependent cell death involved in cancers, including gliomas^10^. The GSE4290 database which has more than 100 samples, was selected and the differential expression of ferroptosis-related genes^7^ in GBM and normal tissues were analyzed. The results showed that FTL gene expression was highest in GBM tissue and was significantly higher compared with the normal group **(Figure 1A and Figure S1B)**. Similar results could be validated in the Rembrandt database which has more than 200 samples **(Figure S1A, C)**. Furthermore, time-dependent ROC was performed to determine the prognostic value of the FTL-based risk score based on the CGGA databases. The AUCs for 3-, 5-, and 10-year overall survival predictions for risk scores were 0.564, 0.569, and 0.557, respectively in the CGGA693 database **(Figure 1B)** and 0.668, 0.678, 0.688, respectively in the CGGA325 database **(Figure 1C)**. Patients from the CGGA325 and CGGA693 databases were classified into high-risk and low-risk groups and the survival curves revealed that a high expression of FTL was associated with poor prognosis in glioma patients **(Figure 1D, E)**.

The specimens of GBM exhibited an extremely high density of cellularity and vascular endothelial hyperplasia, pleomorphic nuclei hyperchromatism and atypical mitosis in HE staining, and abundant SOX2 expression in IHC staining **(Figure 1F)**. IHC staining also displayed a prominent, more intensive FTL expression in GBM tissue compared with that in PT tissue **(Figure 1G and Figure S2A)**, confirmed by FTL protein analysis: a higher protein expression of FTL in GBM tissue compared with that of PT tissue **(Figure 1G)**. M2-like macrophage markers CD163 and IBA1 were upregulated in the high FTL group **(Figure 1F and Figure S2A)**. Moreover, the M1-like macrophage marker IL-1β was downregulated in the high FTL group **(Figure 1F and Figure S2A)**. These results indicated that enhanced FTL was positively correlated with M2 polarization.

**Figure 2:**
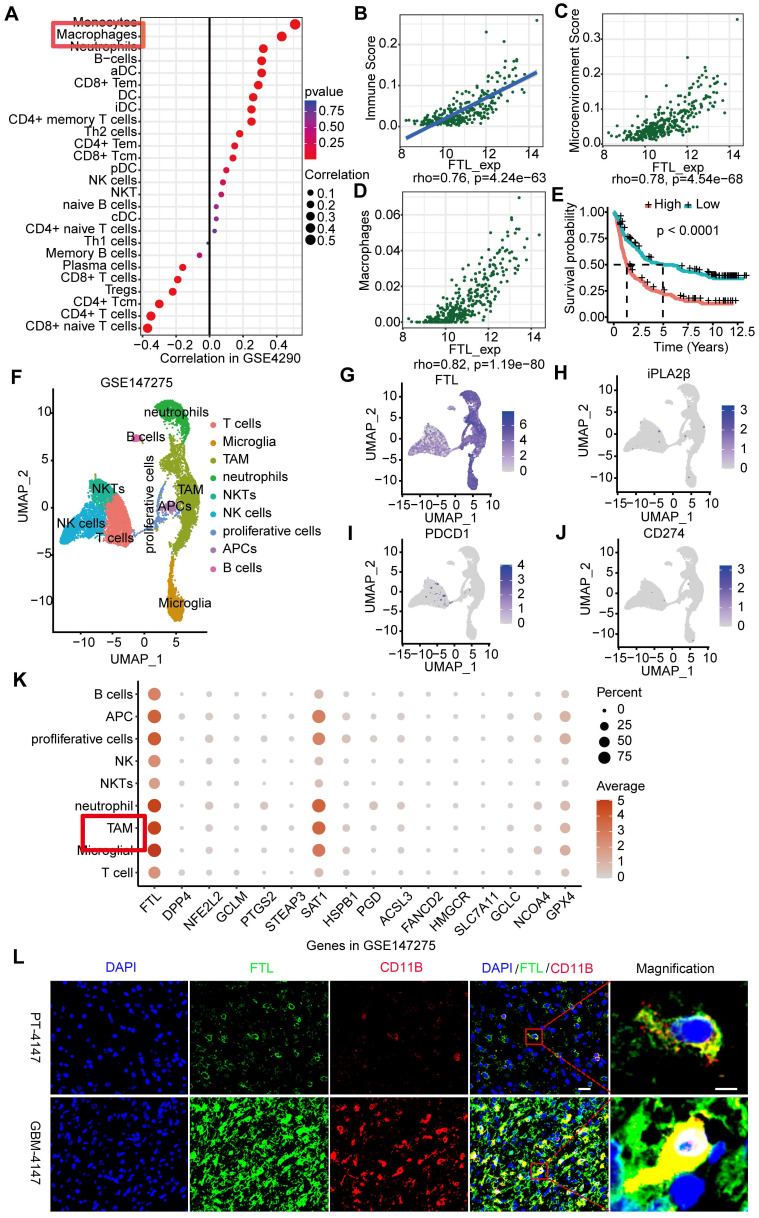
** The role of FTL in the immune microenvironment of glioma. (A)** Immune infiltration cells associated with FTL were performed using the ssGESA algorithm based on the GSE4290 database. **(B)** Correlation analyses of FTL and immune score calculated using ESTIMATE were performed. **(C)** Correlation analyses of FTL and microenvironment scores were performed using Spearman's correlation test. **(D)** Correlation analyses between FTL with macrophages calculated by ESTIMATE, were performed. **(E)** The Kaplan-Meier curve of the immune score on overall survival of CGGA datasets. **(F)** UMAPs of immune constituents from 15 patients with glioma, showing clusters of T cells, microglia, TAM, neutrophils, NKTs, NK cells, proliferative cells, APCs, and B cells with 9 principal components from GSE147275 database. **(G**-**J)** The feature plot of FTL, iPLA2β, PDCD1 and CD274 gene in UMAP. **(K)** The expression of FTL and ferroptosis-related genes in clusters. **(L)** Immunofluorescence images of FTL and CD11B showed that FTL (blue) is co-localized with CD11B. The scale bar in the column represents 20 μm and in the magnification column 5 μm.

To examine the FTL expression at the single cell level, we performed the single-cell analyses of 15 human glioma samples based on the GSE147275, and 9 clusters were identified **(Figure 2F)**: T cells **(Figure S3A)**, microglia **(Figure S3B)**, tumor-associated macrophages (TAMs) **(Figure S3C)**, neutrophils **(Figure S3D)**, NKTs **(Figure S3E)**, NK cells **(Figure S3F)**, proliferative cells **(Figure S3G)**, APCs **(Figure S3H)**, and B cells **(Figure S3I)**. The results showed that FTL was significantly expressed in TAM **(Figure 2G, K)**. In addition, single-cell analyses of glioma samples based on GSE135045 and GSE202371 databases were performed, and FTL was enriched in TAMs **(Figure S4)**. The immunofluorescence staining showed that FTL was co-labeled with TAMs maker (CD11B) and the expressions of FTL and CD11B were higher in GBM tissues than paratumoral tissues **(Figure 2L and Figure S5)**, which further demonstrated that FTL was enriched in TAMs.

### Clinical significance and co-expression network of FTL in glioma

To explore the correlation between FTL and GBM progression, we analyzed FTL expression based on public databases. As shown in **Figure S6A**, FTL expression level was higher in GBM than that in other glioma subtypes and FTL was positively correlated with glioma grade. IDH mutation and 1p/19q co-deletion are unique indicators suggesting a favorable prognosis for glioma patients^11^. We found that FTL expression was significantly higher in IDH1/2 wildtype gliomas and 1p/19q non co-deletion **(Figure S6C, D)**. Further, patients over 42 years old showed higher FTL expression **(Figure S6E)**. In addition, FTL expression was not significantly correlated with gender and progression status **(Figure S6E, F)**.

To investigate the biological function of FTL in glioma, GO and KEGG functional enrichment analyses were conducted to elaborate on the potential biological role of FTL in GBM based on Rembrandt and CGGA325 databases. Biological process (BP) enrichment analysis showed that co-expressed genes of FTL were significantly correlated with the adaptive immune response, regulation of immune effector processes, responses to interferon-gamma, the regulation of leukocyte proliferation, etc. **(Figure S7A)**. Cellular component (CC) analysis showed that co-expressed genes of FTL mainly participated in ficolin-1 rich granule, vesicle lumen, vacuolar lumen, etc. **(Figure S7B)**. Molecular function (MF) enrichment analysis revealed that these genes were significantly involved in immune receptor activity, antigen binding, immunoglobulin receptor binding, etc. **(Figure S7C)**. Co-expressed genes of FTL were significantly associated with immune-associated signaling pathways, such as autoimmune thyroid disease, antigen processing and presentation, etc. via KEGG pathway analysis **(Figure S7D)**. These results suggested that FTL might participate in the network of immunity-related functions in glioma.

### The role of FTL in the immune microenvironment of glioma

The immune microenvironment is an increasingly recognized major cause of cancer immunotherapy. The correlations between FTL and various immune infiltration cells were analyzed through ssGSEA **(Figure 2A, Figure S8A)**; the results showed that FTL was positively correlated with immune infiltration score, microenvironment and macrophages in glioma **(Figure 2B**-**D, Figure S8B**-**D)**. Further, M2 macrophages were positively correlated with FTL in glioma **(Figure S8E)**. We analyzed the association between FTL with immuno-inhibitors and found that IL-10 was significantly associated with FTL expression **(Figure S8F)**. Moreover, there is a positive association between FTL expression and immune checkpoint molecules **(Figure S8G**-**K)**. The prognostic value of the immune infiltration score was analyzed and the results revealed that patients with high immune infiltration scores had poor overall survival **(Figure 2E)**.

### FTL promoted polarization of TAMs into the M2-Like phenotype

**Figure 3:**
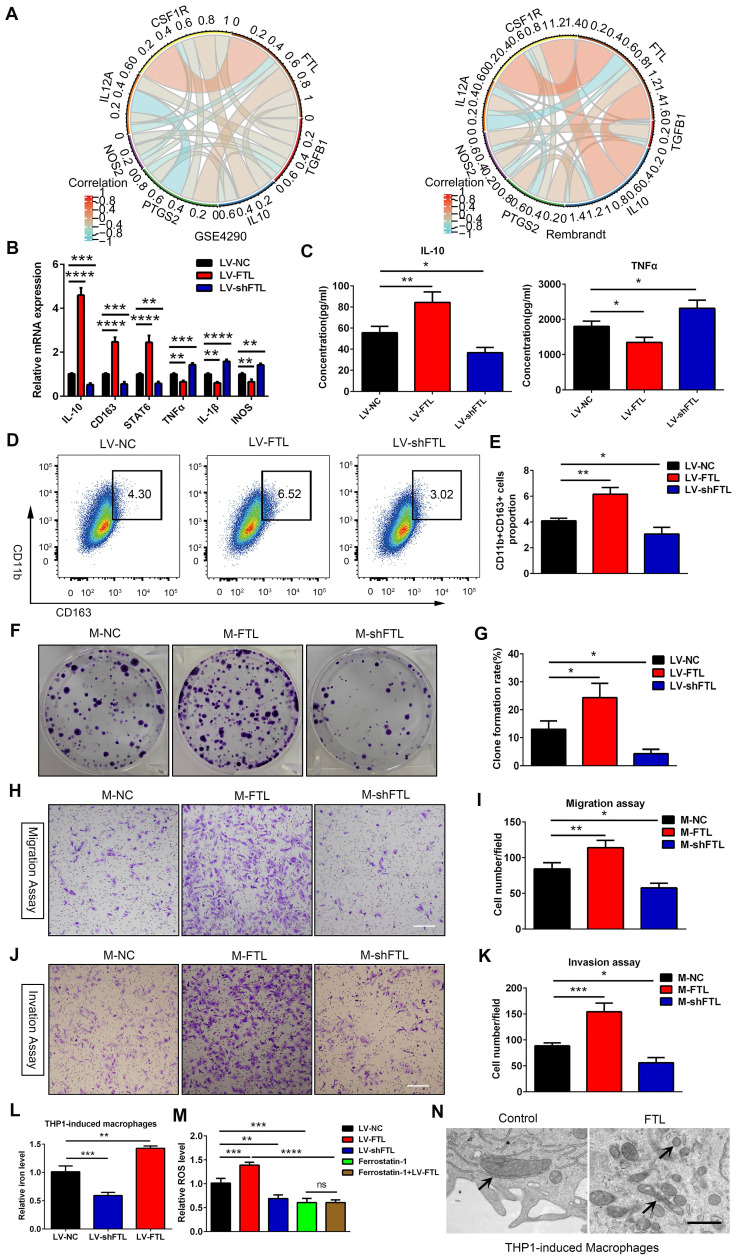
** FTL induced M2 macrophage polarization to promote the progression of glioma cells via reducing iron overload and ROS generation in vitro. (A)** The correlation coefficient between FTL and classical phenotype markers of macrophages in the GSE4290 and Rembrandt databases. **(B, C)** Human THP-1 cells were incubated with PMA (100 ng/ml) for 24 h in vitro to induce them to differentiate into macrophages. Then the macrophages were transfected with LV-NC, LV-FTL or LV-shFTL. The expression levels of CD163, IL-10, STAT6, IL-1β, iNOS and TNFα were determined by qRT-PCR **(B)**. The supernatants of conditioned macrophage transfected with LV-NC, LV-FTL or LV-shFTL for 48h cultures were used to determine the secretion of IL-10 and TNFα by ELISA **(C)**. **(D, E)** Macrophages were treated in the same way as described in B. The induced CD11b^+^CD163^+^ macrophages were determined by flow cytometry **(D)**, and quantification was performed **(E)**. **(F, G)** Clone formation was used to detect the proliferation of MG cocultured with conditioned macrophages transfected with LV-NC (M-NC), LV-FTL (M-FTL) or LV-shFTL (M-shFTL). **(F)**, and quantification was performed **(G)**. **(H, I)** The migration capacity of MG cocultured with conditioned macrophages was examined. Representative images of migratory cells and quantifications are shown (scale bar, 100 μm). **(J, K)** The invasion capacity of MG cocultured with conditioned macrophages was examined. Representative images of invaded cells and quantifications are shown (scale bar, 100 μm). **(L)** Iron content in THP1-induced macrophages transfected with LV-NC, LV-FTL or LV-shFTL. **(M)** ROS content in THP1-induced macrophages transfected with LV-NC, LV-FTL or LV-shFTL. **(N)** Representative images of electron micrograph of THP1-induced macrophages (scale bar, 1 μm).

TAMs are the most abundant tumor-infiltrating immune cells in the glioma TME and initiate antitumor/immunostimulatory (M1) or protumor/immunosuppressive (M2) responses depending on their polarization status^12^. Therefore, we explored whether FTL could change macrophage polarization status. IHC staining showed that the M1 macrophage marker IL-1β was downregulated, while the M2 macrophage marker CD163 and microglial marker IBA1 were elevated in the high FTL group **(Figure 1F and Figure S2A)**. We investigated the relationship between FTL expression and the typical phenotypic macrophage markers for M1 (IL12A, NOS2, PTGS2) and M2 (IL10, TGFB1, CSF1R) by correlation coefficient analyses in the GSE4290 and Rembrandt databases. The changes in major immune cell populations in the glioma microenvironment to FTL enrichment based on Rembrandt and TCGA-glioma databases were analyzed by CIBERSORT. M2 macrophages constitute a high proportion of immune cells in the glioma microenvironment and the proportions of M2-TAMs and neutrophils in high-FTL group was significantly higher than that in low-FTL group **(Figure S9A, D)**. While the proportions of CD8^+^ T cells in the high-FTL group were significantly lower than that in the low-FTL group in the Rembrandt database. There was no significant difference in the proportions of B cells naive, B cells memory, T cells CD4 naive, T cells CD4 memory activated, NK cells resting, NK cells activated, dendritic cells resting and dendritic cells activated between the high-FTL group and the low-FTL group in Rembrandt database **(Figure S9A)**. In the TCGA-glioma database, the proportions of B cells naive and NK cells activated in the high-FTL group were significantly lower than that in the low-FTL group. There was no significant difference in the proportions of B cells memory, T cells CD8, T cells CD4 memory resting and dendritic cells resting between the high-FTL group and low-FTL group in the TCGA-glioma database **(Figure S9D)**. Moreover, FTL expression was positively correlated with M2 macrophage markers **(Figure 3A)**. These results suggested that gliomas with enhanced FTL were characterized by M2 polarization.

To determine whether FTL could induce macrophage polarization toward the M2 phenotype, THP-1-induced macrophages were transfected with LV-NC, LV-FTL and LV-shFTL in vitro. The expression of M2 macrophage markers (CD163, IL-10 and STAT6) and M1 macrophage markers (IL-1β, iNOS and TNFA) were measured by qRT-PCR. Compared with the LV-NC group, the expressions of CD163, IL-10 and STAT6 in the LV-FTL group were significantly increased, while the expressions of IL-1β, iNOS and TNFα were markedly decreased **(Figure 3B)**. Then ELISA was performed to assess the secretion of IL-10 and TNFα in macrophage culture supernatants. FTL significantly increased the secretion of IL-10 in macrophages, while markedly decreasing the secretion of TNFα **(Figure 3C)**. The opposite effect appeared in the shFTL group **(Figure 3B, C)**. Flow cytometry was used to measure the expression of CD163 in macrophages treated with LV-NC, LV-FTL and LV-shFTL. FTL significantly elevated the proportion of CD163^+^ macrophages **(Figure 3D, E)**. Furthermore, we performed a coculture system in vitro to explore the effect of macrophages treated with FTL on glioma progression. GBM-Z1 glioma cells cultured in the supernatant of macrophages transfected with LV-FTL elevated the clone formation number compared with that in the LV-NC group **(Figure 3F, G)**. Migration and invasion assays confirmed that FTL-treated macrophages significantly increased the motility of glioma cells **(Figure 3H**-**K)**.

**Figure 4:**
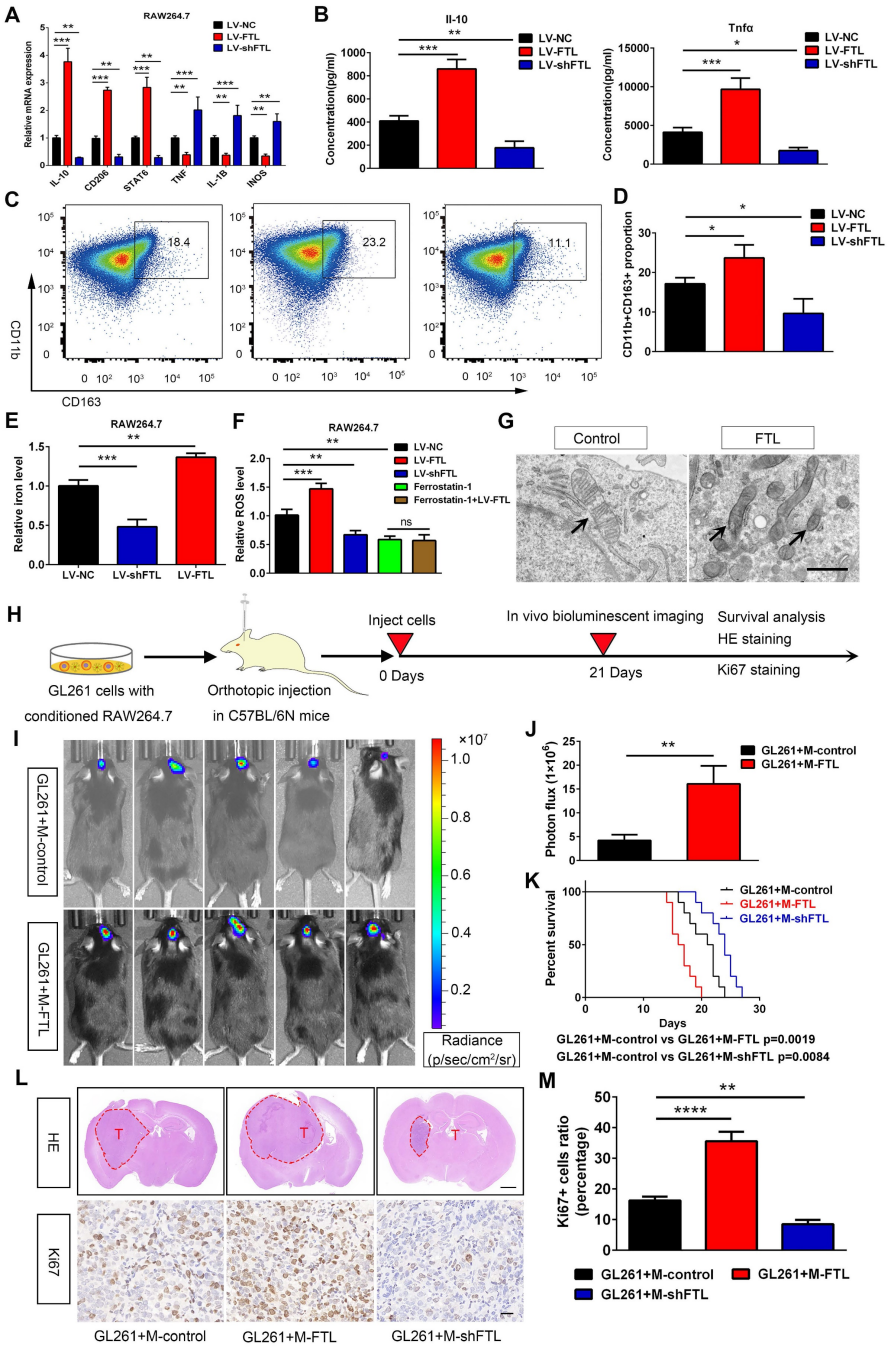
** FTL induced M2 macrophage polarization to promote the progression of murine GL261 gliomas via reducing iron overload and ROS generation. (A)** The RAW264.7 macrophages were transfected with NC, LV-FTL or LV-shFTL. The expression levels of IL-10, CD206, STAT6, TNFα, IL-1β, INOS were determined by qRT-PCR. **(B)** The supernatants of RAW264.7 macrophage transfected with LV-NC, LV-FTL or LV-shFTL for 48h were used to determine the secretion of Il-10 and Tnfα by ELISA. **(C, D)** Macrophages were treated in the same way as described in A. The CD11b^+^CD163^+^ macrophages were determined by flow cytometry **(C)**, and quantification was performed **(D)**. **(E)** Iron content in RAW264.7 macrophages transfected with LV-NC, LV-FTL or LV-shFTL. **(F)** ROS content in RAW264.7 macrophages transfected with LV-NC, LV-FTL or LV-shFTL.** (G)** Representative images of electron micrograph of RAW264.7 cells (scale bar, 1 μm). **(H)** Scheme of experimental design for the role of FTL on macrophage polarization induced and glioma progression. **(I, J)** In vivo bioluminescent imaging analysis of tumor growth in xenograft C57BL/6N mice bearing GL261 glioma cells with control-macrophages (M-control) or FTL-macrophages (M-FTL). Representative images on day 21 post-implantation are shown **(I)**, and quantification was performed **(J)**. **(K)** Survival analysis of animals implanted with GL261 glioma cells with M-control or M-FTL. **(L)** Representative images of HE staining (up column) and IHC staining for Ki67 of sections from xenograft mouse brains with GL261 and M-control, M-FTL or M-shFTL. The scale bar in up column represents 1000 μm and in low column 20 μm. **(M)** Quantification of Ki67 staining in sections from xenograft mouse brains with GL261 and M-control, M-FTL or M-shFTL.

In addition, we also explored the role of FTL on RAW264.7 macrophage polarization. FTL markedly increased the gene expressions of CD206, IL-10 and STAT6, which significantly decreased the expressions of TNFα, IL-1β and INOS in RAW264.7 macrophages **(Figure 4A)**. Then the ELISA assay showed that FTL markedly increased the secretion of IL-10 in macrophages, and significantly decreased the secretion of TNFα **(Figure 4B)**. Next, flow cytometry analysis also showed that FTL significantly increased the proportion of CD163^+^ macrophages **(Figure 4C, D)**. Opposite results occurred in the LV-shFTL group **(Figure 4A**-**D)**.

To validate the role of FTL on M2 macrophage polarization in vivo, macrophages transfected with LV-control (M-control), LV-FTL (M-FTL) and LV-shFTL (M-shFTL) were co-implanted with GL261 glioma cells in situ in C57BL/6N mice. In vivo bioluminescent imaging results showed the mice in the M-FTL group had stronger signals than the M-control mice at three weeks after implantation **(Figure 4I, J)**. Moreover, survival analyses revealed that the mice in the M-FTL group had a significantly shorter survival time than those in the control groups **(Figure 4K)**. Opposite results occurred in the M-shFTL group **(Figure S10 and Figure 4K)**. HE and ki67 staining showed that C57BL/6N mice implanted with GL261 glioma cells and M-FTL had a higher proliferative capacity of glioma cells **(Figure 4L, M)**. Opposite results occurred in the M-shFTL group **(Figure 4L, M)**.

Ferroptosis is an iron-dependent cell death, characterized by the accumulation of lipid peroxidation products and ROS involved in cancers^10^. The correlation between ferroptosis and macrophage polarization based on Rembrandt and TCGA-glioma databases by ssGSEA was analyzed, and the results showed that ferroptosis was significantly correlated with macrophage polarization (R = 0.84, 0.86) **(Figure S11)**. Electron micrographs showed that macrophages overexpressing FTL showed significant morphological characteristics of ferroptosis, exhibiting a condensed mitochondrial membrane with a ruptured outer mitochondrial membrane and the disappearance of mitochondrial cristae **(Figure 3N and Figure 4G)**. Intracellular Fe^2+^ levels were found to be increased in the FTL overexpressing groups **(Figure 3L, 4E)**. In addition, FTL dramatically increased ROS levels in THP-1 and RAW264.7 macrophages through DCFHDA staining **(Figure 3M, 4F)**. Ferroptosis inhibitor, ferrostatin-1 dramatically decreased ROS levels in THP-1 and RAW264.7 macrophages **(Figure 3M, 4F)**. Compared with the ferrostatin-1 group, there was no significant difference in ROS levels in the ferrostatin-1+LV-FTL group **(Figure 3M, 4F)**, suggesting that FTL promoted the ferroptosis pathway by inducing iron overload and ROS generation.

Taken together, these results indicated that FTL could induce M2 macrophage polarization and promote glioma progression by inducing iron overload and ROS generation.

### FTL inhibited iPLA_2_β in TAMs

**Figure 5:**
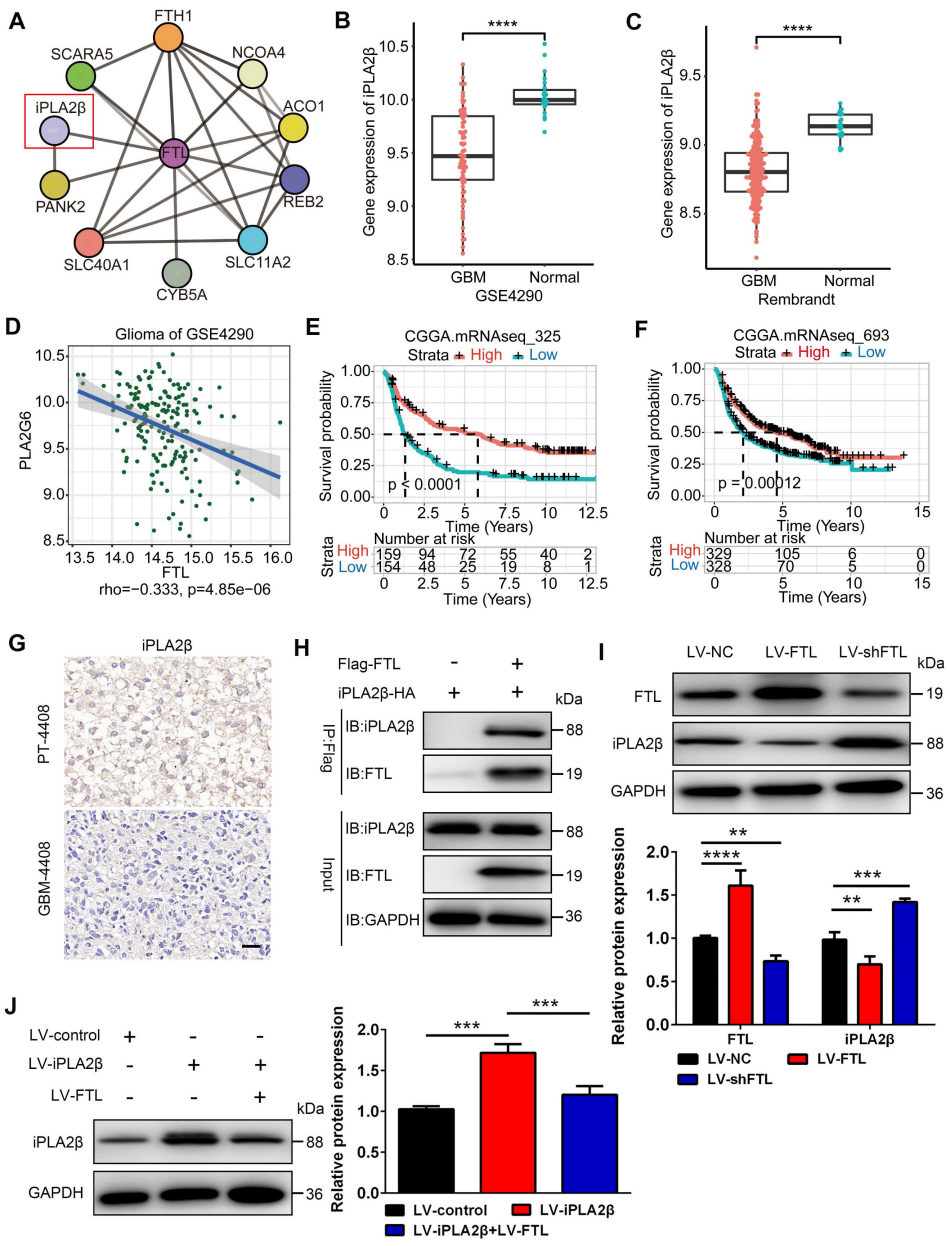
** FTL inhibited iPLA2β in TAMs. (A)** Potential FTL interactors were predicted using the STRING database. **(B, C)** The expression of iPLA2β was compared between normal and GBM tissue in GSE4290 **(B)** and Rembrandt databases **(C)**. **(D)** Correlation analyses of FTL and iPLA2β were performed using Spearman's correlation test based on the GSE4290 database. **(E, F)** The correlation of iPLA2β expression level with survivals from the CGGA database. **(G)** IHC images of iPLA2β expression in GBM-4408 and PT-4408 (scale bar, 20 μm). **(H)** FTL co-immunoprecipitates with iPLA2β, thus confirming the interaction predicted using STRING. **(I)** The protein expression of FTL and iPLA2β relative to GAPDH in TAMs transfected with LV-NC, LV-FTL or LV-shFTL was examined by western blot.** (J)** The protein expression of iPLA2β relative to GAPDH in TAMs transfected with LV-control, LV-iPLA2β only or with LV-FTL was examined by western blot.

To explore how FTL modulates M2 macrophage polarization, we used the STRING database to screen for molecules that could potentially interact with FTL and identified Ca^2+^-independent phospholipase A_2_β (iPLA_2_β), which is associated with macrophage polarization^13^, as a putative candidate **(Figure 5A)**. In the GSE4290 and Rembrandt databases, the expression of iPLA_2_β was found to be significantly lower in GBM than in normal tissues **(Figure 5B, C)**, which was negatively correlated with the expression of FTL **(Figure 5D)**. Moreover, single-cell analysis based on the GSE147275 database showed that iPLA_2_β was lowly expressed in TAM **(Figure 2H)**. IHC staining showed that iPLA_2_β was downregulated in GBM compared with PT **(Figure 5G)**. The survival curves revealed that the high expression of iPLA_2_β was correlated with a better prognosis of glioma patients based on the CGGA325 and CGGA693 databases **(Figure 5E, F)**. To confirm whether FTL interacts with iPLA_2_β, a CO-IP experiment was performed in RAW264.7 macrophages, and the results showed that FTL indeed interacted with iPLA_2_β **(Figure 5H)**. Western blot results showed that overexpressed FTL decreased iPLA_2_β expression **(Figure 5J)**, while FTL knockdown increased iPLA_2_β expression, suggesting that iPLA_2_β was involved in this regulation **(Figure 5I)**. Moreover, the overexpression of iPLA_2_β could be rescued by FTL **(Figure 5J)**. These results revealed that FTL inhibited the expression of iPLA_2_β in TAMs.

### FTL promotes M2 macrophage polarization and glioma angiogenesis in vivo

**Figure 6:**
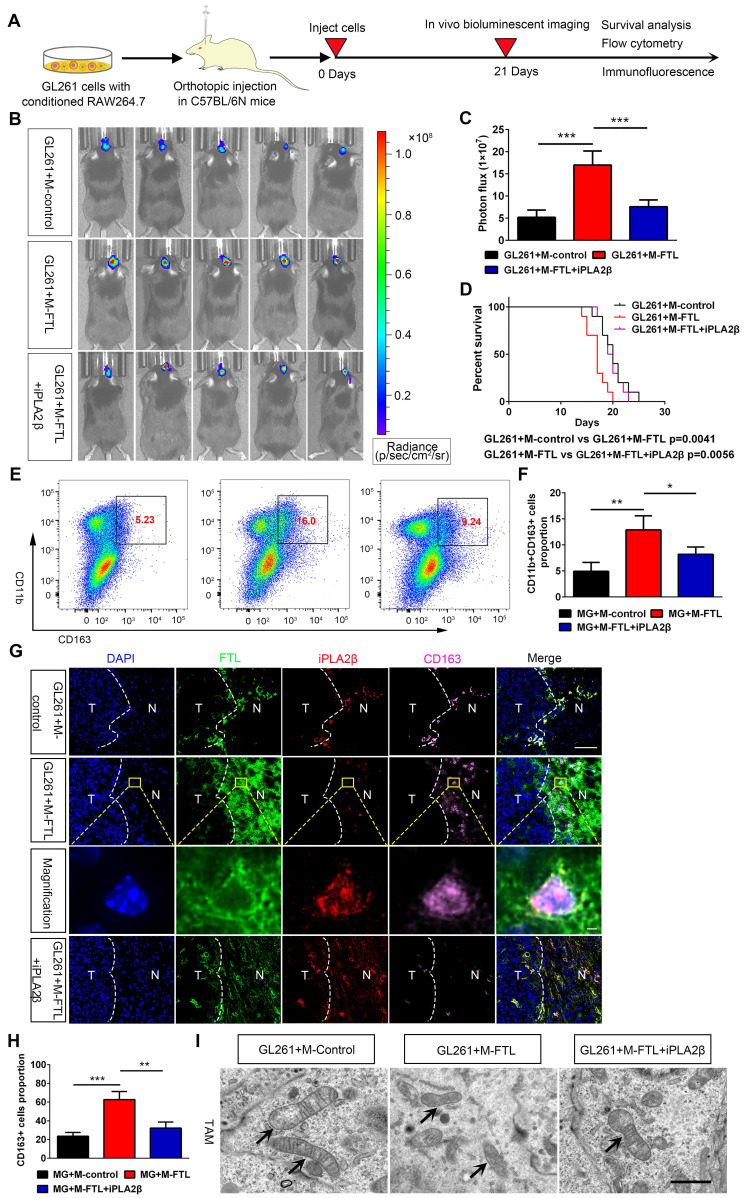
**FTL inhibited M2 macrophage polarization and promoted glioma in vivo. (A)** Scheme of experimental design showing the role of FTL in vivo. **(B)** In vivo bioluminescent imaging analysis of tumor growth in xenograft C57BL/6N mice bearing GL261 glioma cells with M-control, M-FTL or M-FTL+iPLA2β. Representative images of day 21 post-implantation are shown, and quantification was performed **(C)**. **(D)** Survival analysis of animals implanted with GL261 glioma cells with M-control, M-FTL or M-FTL+iPLA2β. **(E, F)** The CD11b^+^CD163^+^ macrophages were determined in xenograft C57BL/6N mice bearing GL261 glioma cells with M-control, M-FTL or M-FTL+iPLA2β by flow cytometry **(E)**, and quantification was performed **(F)**.** (G)** Immunofluorescence images of FTL, iPLA2β and CD163 in xenograft C57BL/6N mice bearing GL261 glioma cells with M-control, M-FTL and M-FTL+iPLA2β. M2 macrophage cells (pink, CD163) are accumulated in peritumoral regions and co-localized with FTL (blue) and iPLA2β (red) in xenograft C57BL/6N mice bearing GL261 glioma cells with M-FTL. The scale bar in the column represents 50 μm and in the magnification column 2 μm.** (H)** Quantification of CD163^+^ cells was performed. Units represent the % of voxels in the CD163 channel colocalized with DAPI. **(I)** Representative images of electron micrograph in xenograft C57BL/6N mice bearing GL261 glioma cells with M-control, M-FTL or M-FTL+iPLA2β (scale bar, 1 μm).

To verify the mechanism of FTL on M2 macrophage polarization in vivo, macrophages transfected with lentivirus overexpressing FTL or with iPLA_2_β were co-implanted with GL261 glioma cells into C57BL/6N mice to establish orthotopic xenografts. Bioluminescent imaging revealed that mice implanted with GL261 and FTL-overexpressing macrophages had stronger signals compared with the control group **(Figure 6B)**. Moreover, C57BL/6N mice implanted with GL261 and FTL+iPLA_2_β overexpressing macrophages reversed the bioluminescence signals of the FTL overexpressing macrophages **(Figure 6B, C)**. The mice implanted with GL261 and FTL-overexpressing macrophages had a shorter survival time **(Figure 6D)**. C57BL/6N mice implanted with GL261 and FTL+iPLA_2_β overexpressing macrophages had better survival compared with the FTL overexpressing groups **(Figure 6D)**. Flow cytometry analysis showed that FTL significantly promoted CD163 expression in mice implanted with GL261 and FTL-overexpressing macrophages. Moreover, C57BL/6N mice implanted with GL261 and FTL+iPLA_2_β overexpressing macrophages had a lower CD163^+^ cell proportion compared with the FTL overexpressing groups **(Figure 6E, F)**. Immunofluorescence images using CD163 staining showed that M2 macrophages accumulated at the tumor border **(Figure 6G)**. FTL and iPLA_2_β expression were expressed in peri-tumoral tissue and co-localized with M2 macrophages **(Figure 6G)**. Mice implanted with GL261 and FTL-overexpressing macrophages had a higher CD163^+^ cell proportion compared with the control groups **(Figure 6G, H)**. C57BL/6N mice implanted with GL261 and FTL+iPLA_2_β overexpressing macrophages had a lower CD163^+^ cell proportion compared with the FTL overexpressing groups **(Figure 6G, H)**. Mice implanted with GL261 and FTL-overexpressing macrophages showed significant morphological characteristics of ferroptosis, exhibiting a condensed mitochondrial membrane with a ruptured outer mitochondrial membrane and the disappearance of mitochondrial cristae **(Figure 6I)**. C57BL/6N mice implanted with GL261 and FTL+iPLA_2_β overexpressing macrophages reversed the morphological changes by FTL overexpressing **(Figure 6I)**.

Flow cytometry analysis was also performed to evaluate the effect of FTL on major immune cell populations in the glioma microenvironment in vivo. FTL reduced the proportions of CD8^+^ T cells **(Figure S12B)**, B cells **(Figure S12E)** and NK cells **(Figure S12G)** in mice implanted with GL261 and FTL-overexpressing macrophages, while significantly increasing the proportions of neutrophils **(Figure S12I)**. Moreover, C57BL/6N mice implanted with GL261 and FTL+iPLA_2_β overexpressing macrophages had higher proportions of CD8^+^ T cells **(Figure S12B)**, B cells **(Figure S12E)** and NK cells **(Figure S12G)**, and lower proportions of neutrophils compared with the FTL overexpressing groups **(Figure S12I)**. In addition, there was no significant difference in the proportions of M1 **(Figure S12K)** and CD4^+^ T cells **(Figure S12A)** among the three groups.

The cytokines levels to FTL enrichment based on Rembrandt and TCGA-glioma databases were analyzed, and the gene expressions of IL-10 and TGFB1 in the high-FTL group was significantly higher than that in the low-FTL group **(Figure S9B, C, E, F)**, while the gene expressions of IL-12A in the high-FTL group was significantly lower than that in the low-FTL group in the TCGA-glioma database **(Figure S9B, C)**. Flow cytometry analysis was performed to measure the cytokines levels, intracellular staining suggested that FTL increased the proportions of IL-2 in CD4^+^ T cells **(Figure S12B)**, TNFα in CD8^+^ T cells **(Figure S12D)**, IL-10 in B cells **(Figure S12F)** and M2 macrophages **(Figure S12M)** in mice implanted with GL261 and FTL-overexpressing macrophages, while reduced the proportions of IFN-r in NK cells **(Figure S12H)**, and IL-12 in neutrophils **(Figure S12J)** and M1 macrophages **(Figure S12L)**. C57BL/6N mice implanted with GL261 and FTL+iPLA_2_β overexpressing macrophages had lower proportions of IL-2 in CD4^+^ T cells **(Figure S12B)**, TNFα in CD8^+^ T cells **(Figure S12D)**, IL-10 in B cells **(Figure S12F)** and M2 macrophages **(Figure S12M)**, and higher proportions of IFN-r **(Figure S12H)** in NK cells, and IL-12 in neutrophils **(Figure S12J)** and M1 macrophages **(Figure S12L)** compared with the FTL overexpressing groups. IL-10^14^ and TGFB1^15^ play as important driving forces of tumor immune escape and contribute to the immunosuppressive tumor microenvironment of glioblastoma. IL-12 has been demonstrated to play the immunological antitumor effect on high-grade glioma^16^. These results suggested that FTL in TAMs induced M2 macrophage polarization and reduced the proportions of CD8^+^ T cells, B cells and NK cells by iPLA_2_β.

**Figure 7:**
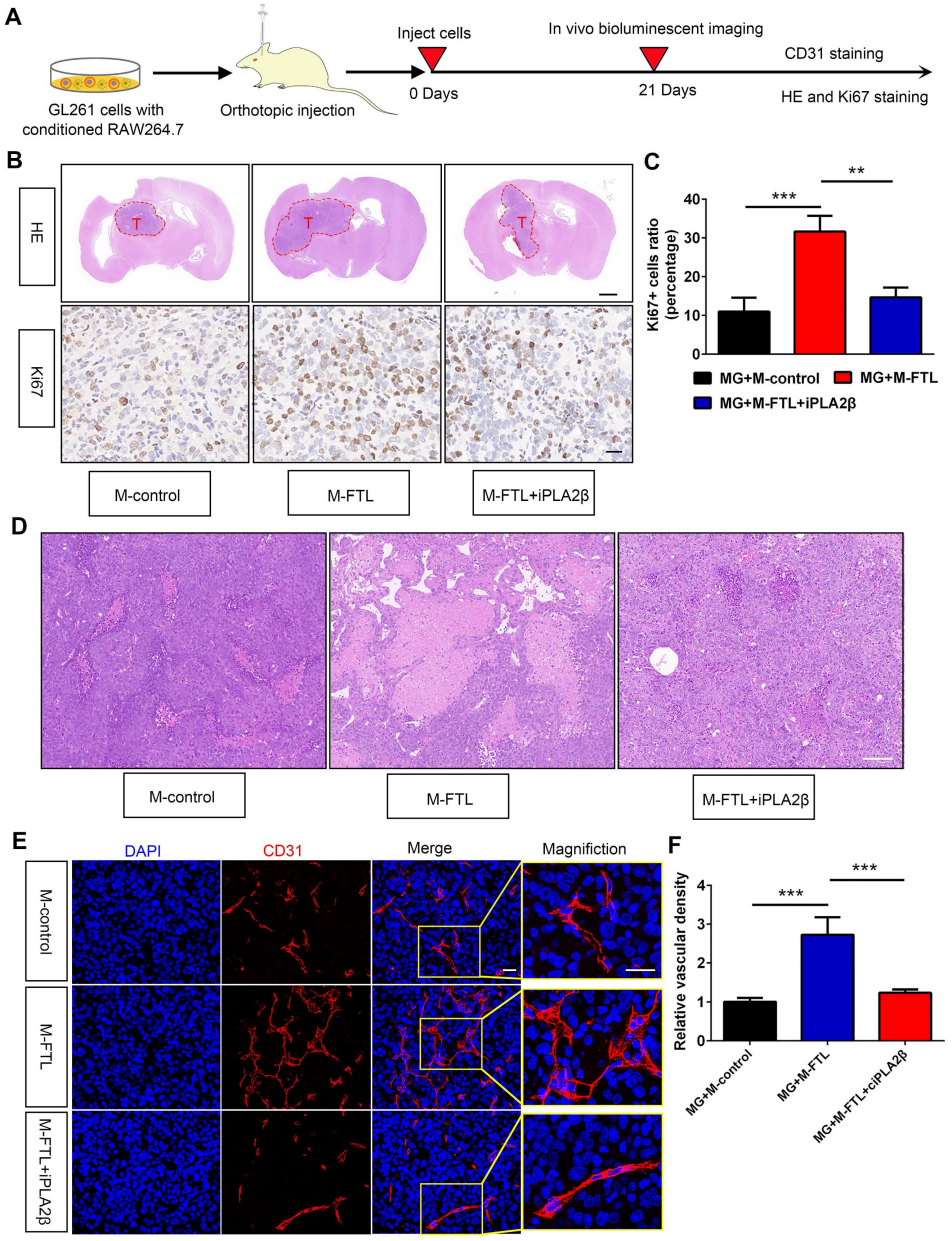
**FTL promoted glioma vascularization and growth in vivo. (A)** Scheme of experimental design showing the role of FTL on glioma vascularization and growth in vivo. **(B)** Representative images of HE staining and IHC staining for Ki67 of sections from xenograft mouse brains with GL261 and M-control, M-FTL or M-FTL+iPLA2β. The scale bar in the up column represents 1000 μm and in the low column 20 μm. **(C)** Quantification of Ki67 staining in sections from xenograft mouse brains with GL261 and M-control, M-FTL or M-FTL+iPLA2β. **(D)** Tumor sections were stained with HE and imaged (scale bar, 100 μm). **(E)** Immunofluorescence images of CD31 in xenograft C57BL/6N mice bearing GL261 glioma cells with conditioned RAW264.7 macrophages. The right panel shows the magnification. Both scale bars represent 20 μm.** (F)** Quantitative analysis of relative vascular density by CD31 staining.

HE and IHC staining showed that glioma cells from C57BL/6N mice implanted with GL261 and FTL-overexpressing macrophages had a higher proliferative capacity **(Figure 7B, C)**. Moreover, mice implanted with GL261 and FTL+iPLA_2_β overexpressing macrophages reversed the proliferative capacity in the FTL overexpressing group **(Figure 7B, C)**. To explore whether FTL regulates glioma angiogenesis, we analyzed GL261 tumor vasculature by CD31 staining. The tumor vessels of control mice showed the typical morphological characteristics of human GBM vascular abnormality, i.e. tortuous and dilated with extensive necrosis. The vessels in the mice implanted with GL261 and M-FTL appeared abnormal exhibiting more tortuous vessels **(Figure 7D)** with more extensive necrosis, a defining pathological feature of GBM, and more importantly, significantly increased vascular density in tumors **(Figure 7E)**. The blood vessels in the mice implanted with GL261 glioma cells and M-FTL+iPLA_2_β appeared partially normalized, as evidenced by lesser tortuous vessels **(Figure 7D)** with minimal necrosis, and significantly reversed vascular density in the tumors **(Figure 7E)**. Based on these results, FTL significantly promotes M2 macrophage polarization and tumor angiogenesis in glioma.

### FTL inhibition of TAMs enhanced sensitivity to anti-PD1 therapy in glioma

**Figure 8:**
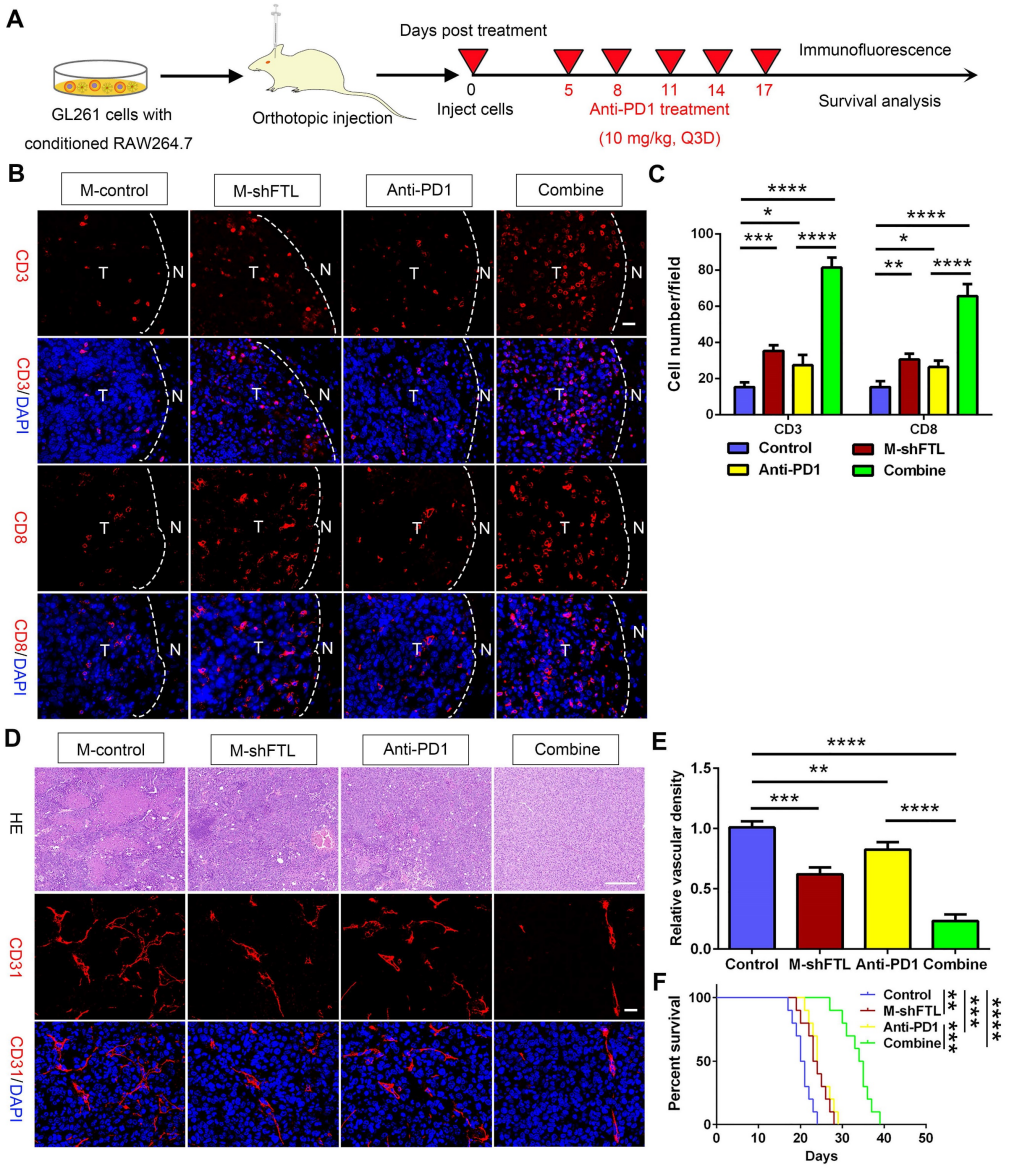
** FTL inhibition sensitized GBM to anti**-**PD1 immunotherapy. (A)** Schematic illustration of the experimental design of combination M-shFTL with anti-PD1 in GL261-bearing C57BL/6N mice. **(B)** Immunofluorescence of CD3 and CD8 in tumors from GL261-bearing C57BL/6N mice under different treatments (scale bar, 20 μm). **(C)** Quantitative analysis of CD3 and CD8 in xenograft C57BL/6N mice bearing GL261 glioma cells under different treatments (n = 3). **(D)** Immunofluorescence of CD31 and HE in tumors from GL261-bearing C57BL/6N mice under different treatments. The scale bar in the up column represents 100 μm and in the low column 20 μm. **(E)** Quantitative analysis of relative vascular density by CD31 staining. **(F)** Survival analysis of GL261-bearing C57BL/6N mice under different treatments (n = 10).

Facilitating the transition from an immunosuppressive TME to an immune-supported TME can improve the effectiveness of cancer immunotherapy^17^, suggesting that the effectiveness of immune checkpoint blockade may be increased by inhibiting FTL. To validate this hypothesis, we first analyzed the gene expression levels of PD1 (PDCD1 gene) and PDL1 (CD274 gene) using single-cell sequencing based on the GSE147275 database. The gene expression of PDCD1 and CD274 were mainly located in the T cell cluster in glioma **(Figure 2I, J)**. Then we orthotopically injected GL261 glioma cells and macrophages transfected with LV-shFTL into C57BL/6N mice and then conducted a PD1 mAb injection to assess the anti-tumor capability **(Figure 8A)**. FTL inhibition increased the number of CD3^+^ T cells and CD8^+^ T cells **(Figure 8B, C)**. The combined group had a significantly increased CD3^+^ T cell and CD8^+^ T cell abundance compared with the anti-PD1 treatment group **(Figure 8B, C)**. These results indicated that T-cell recruitment is improved in gliomas of mice implanted with GL261 and M-shFTL, which is beneficial for PD-1 blocking immunotherapy.

The M-shFTL or anti-PD1 treated mice had less tortuous vessels and necrosis, and more importantly, significantly decreased vascular density in tumors **(Figure 8D, E)**. Compared with the anti-PD1 group, the vessels in the mice of the combined group appeared partially normalized, with lesser tortuous vessels and minimal necrosis, and the vascular density in the tumors was significantly reduced **(Figure 8D, E)**. M-shFTL and anti-PD1 treatment improved survival with a median survival of 23.5 and 24 days, respectively, compared with a median survival of 20.5 days in control groups **(Figure 8F)**. Moreover, compared to 24 days anti-PD1 group, the median survival of anti-PD1 combined with M-shFTL was further prolonged to 34.5 days **(Figure 8F)**. We therefore tentatively conclude that FTL inhibition facilitated the immune-supported microenvironment and enhanced glioma sensitivity to anti-PD1 therapy.

## Discussion

The brain TME, which is highly immunosuppressive and closely involved in glioma progression, is enriched with TAMs^18^. Previous studies have investigated the role of FTL in promoting glioma progression^19^. However, few studies have focused on its effect on the glioma TME, a perspective emphasized in the present study. Our study showed that FTL facilitated the development of a protumor microenvironment by promoting M2 polarization of TAMs. We further demonstrated that TAMs overexpressing FTL significantly promoted the proliferation, invasion and migration of glioma cells. Accumulating evidence indicates that TAMs promote glioma progression and result in poor prognosis by acquiring an M2-like phenotype^20^. Several mediators produced by M2-TAMs, such as epidermal growth factor (EGF)^21^ and stress-inducible protein 1^22^, promoted GBM invasion and expansion. In addition, some mediators released by tumor cells trigger TAMs to support GBM growth^23^. For instance, CCL2 released by GBM cells is not only a chemoattractant for TAMs but also binds to the CCL2 receptor expressed on TAMs^24^. CCL2 triggers TAM secretion of IL-6, which reciprocally promotes GBM progression. CCL2 triggers TAM secretion of IL-6, which reciprocally promotes GBM progression. These findings imply that FTL plays a significant role in TAMs promoting glioma progression.

Our analysis of the GSE4290 and Rembrandt databases, as well as our preclinical tests, revealed a significant upregulation of FTL in GBM. FTL was shown as an indicator of poor prognosis and was positively associated with clinical characteristics of malignancy including IDH wild type and 1p/19q non co-deletion. Furthermore, we demonstrated that FTL was significantly upregulated in GBM. These results provide a perspective for FTL as a prognostic indicator of GBM.

Immune cells, chemokines and checkpoints are recognized as biomarkers of immune evasion and tumor progression^25^. TAMs enriched in the glioma TME can secrete cytokines such as IL-10 and TGFβ, which decrease the activities of immune cells and promote glioma progression^26^. Our study demonstrated that FTL may be involved in neutrophil-related immunity, adaptive immune responses and cytokine binding. Moreover, the upregulation of FTL was positively associated with a high immunoinfiltration score, microenvironment, macrophages, and M2 macrophages in glioma. FTL expression was positively correlated with the levels of the immunoinhibitory factors associated with the glioma TME. The CIBERSORT of major immune cell populations in glioma microenvironment to FTL enrichment showed that neutrophils in the high-FTL group was significantly higher than that in the low-FTL group. While the proportions of CD8^+^ T cells in the high-FTL group were significantly lower than that in the low-FTL group. These data suggest that FTL may participate in glioma immunosuppression and may serve as a potential therapeutic target for glioma immunotherapy.

TAMs were polarized into antitumor/immunostimulatory (M1) or protumor/immunosuppressive (M2) phenotypes under the influence of the TME^27^. Our results of single-cell analysis and immunofluorescence staining demonstrated that FTL was enriched in TAMs. TAMs account for the majority, rendering an immunosuppressive TME. IL-1β, TNFα and iNOS were identified as markers of M1 macrophages that inhibited tumor progression. CD163, CD206, IL-10 and STAT6 are regarded as markers of M2 macrophages that promoted tumor progression^28^, suggesting that targeting M2 macrophages in the glioma TME may be an alternative therapeutic strategy. We transfected LV-shFTL into THP-1-induced macrophages and RAW264.7 macrophages to specifically knock down the expression of FTL in macrophages and found that FTL could promote M2 macrophage polarization, and that FTL inhibition could reprogram tumor-promoting macrophages by inhibiting M2 macrophage polarization, thus inhibiting the glioma progression, suggesting that targeting FTL might become a promising immunotherapy strategy for glioma.

Ferroptosis is an iron-dependent programmed cell death newly discovered by Dixon^29^ involved in cancers by the accumulation of lipid peroxidation products and ROS^30^. Recent studies have shown that ferroptosis was involved in polarization of tumor-associated macrophages and played diverse effects on macrophage polarization in different circumstances. Ferroptosis can polarize M2-type macrophages into M1-type, thereby enhancing anti-tumor immunity in hepatocellular carcinoma^31^. However, in non-small cell lung cancer, ferroptosis is responsible for the polarization and recruitment of M2-TAMs via ROS/PI3K^32^. Autophagy-dependent ferroptosis drives macrophages to switch to an M2-like protumor phenotype via activation of oncogenic KRAS^33^. In glioma, ferroptosis promoted the recruitment and polarization of TAMs into the M2-like phenotype^34^. This might be due to tissue-specific or cancer-specific effects^35^. In our study, the correlation between ferroptosis and macrophage polarization based on Rembrandt and TCGA-glioma databases by ssGSEA was analyzed, and the results showed that ferroptosis was significantly correlated with macrophage polarization. FTL enrichment in TAMs promoted polarization of TAMs into M2-like phenotype by activating ferroptosis pathways. Further efforts are still required to achieve a more comprehensive understanding of ferroptosis on macrophage polarization.

ROS is the key executor of oxidative stress, which causes macrophage ferroptosis^10^. Iron overload can induce ROS by the Fenton reaction, which leads to cell death^36^, which could affect the polarization and recruitment of macrophages. However, iron was a double-edged sword in regulating macrophage polarization. Several studies showed that iron enhanced M1-like macrophage polarization and inhibited M2-like macrophage polarization^37^. However, iron also could promote M2-like macrophage polarization and inhibit M1-like macrophage polarization. In IFN-γ-stimulated RAW264.7 cells, exogenous iron supplementation lowers the expression of M1 markers^38^. Exogenous iron supplementation and endogenic iron-rich human dermal fibroblasts extracellular matrix both enhance THP-1 cells polarizing into M2 phenotype^39^. Mechanistically, iron shapes macrophage polarization via cellular signaling pathways, cellular metabolism, and epigenetic regulation^40^. The differences in the direction of macrophage polarization reflect that the mechanisms underlying iron in shaping macrophage polarization are complex and diverse, and the specific mechanisms remain to be explored. Our study demonstrated that FTL induced M2 macrophage polarization by increasing iron overload and ROS via inhibiting iPLA_2_β. iPLA_2_β has previously been reported to favor an M1 macrophage phenotype^13^, and suppress ferroptosis by removing oxidized PUFA tails from PL^41^. We concluded that FTL promotes M2 macrophage polarization via promoting the ferroptosis pathway by inhibiting iPLA2β; this polarization of macrophages to the M2 type promotes glioma progression.

In addition to TAMs, this study also reported the effect of FTL on T cells in the glioma TME. The relative proportions of CD3^+^ T and CD8^+^ T cells in M-shFTL groups were increased compared with those in the control group, suggesting that M-shFTL improved T-cell recruitment in glioma. During the anti-tumor immune response, CD8^+^ T cells released cytotoxic molecules to specifically recognize and kill tumor cells. FTL inhibition reprogrammed the immune microenvironment by triggering the activation of CD8^+^ T cells, thus enhancing anti-tumor immunity. Moreover, FTL inhibition significantly improved the therapeutic effect of PD1 mab on glioma. These results suggested that inhibition of FTL in TAMs sensitized glioma to anti-PD1 therapy.

Angiogenesis is a hallmark of the occurrence and progression of solid tumors^42^. GBM is characterized by angiogenesis in forming new vascular networks towards novice tumors, which are required for tumor formation and proliferation^43^. TAMs in glioma have been shown to release VEGF-A, which stimulates tumor angiogenesis^43^. TAMs could enhance the vascular mimicry of glioma cells by secreting cytokines IL-6^44^. TAM-induced MMP-2 and MMP-9 could promote angiogenesis and tumor vascularization^45^. Our study demonstrated that inhibiting FTL in TAMs attenuates tumor angiogenesis. We concluded that FTL regulated glioma angiogenesis by inducing macrophage polarization to M2-like phenotype. However, the mechanism of FTL in glioma angiogenesis requires further exploration.

In present study, we reported FTL could enhance immunosuppressive TME by promoting M2 macrophage polarization, thereby facilitating the malignant progression of glioma cells. Our study supports targeting FTL in combination with immune-checkpoint blockade therapy, which is expected to establish efficient therapeutic options for FTL intervention in glioma immunotherapy.

## Conclusion

In summary, the present study revealed that inhibition of FTL reprogrammed the TME by inhibiting M2 macrophage polarization and triggering the activation of CD8^+^ T cells. Combining anti-PD1 immunotherapy with FTL inhibition may improve disease response in glioma patients and requires further investigation.

## Supplementary Material

Supplementary figures and tables.

## Abbreviations

TME: tumor microenvironment
GBM: glioblastoma
TAMs: tumor-associated macrophages
FTL: ferritin light chain
ROS: reactive oxygen species
PT: para-tumor tissue
DMEM: Dulbecco's modified Eagle's medium
ROC: receiver operating characteristics
PFA: paraformaldehyde
DAPI: 4′,6′-diamidino-2-phenylindole
IHC: immunohistochemical
HRP: horseradish peroxidase
IP: immunoprecipitation
BP: biological process
CC: cellular component
MF: molecular function
iPLA_2_β: Ca^2+^-independent phospholipase A_2_β
