# Supplementary material for: Ferritin light chain promotes the reprogramming of glioma immune microenvironment and facilitates glioma progression
Source: Theranostics. 2023 Jun 26;13(11):3794–813. doi: 10.7150/thno.82975 (PMC10334844; doi:10.7150/thno.82975)
Supplement: Supplementary file 1 — Supplementary figures and tables. [file thnov13p3794s1.pdf]

Ferritin light chain promotes the reprogramming of glioma immune microenvironment and facilitates glioma progression

Hong-jiang Li, Chao Yang, Yan-fei Wei, Xue-yuan Li, Wei Jiang, Yi-ran Xu, Li-feng Li, Rong-qun Guo, Di Chen, Peng Gao, Hao-hao Zhang, Hui Qin, Zhen-yu Zhang, Xian-zhi Liu, Dong-ming Yan

Supplementary Figures and Tables

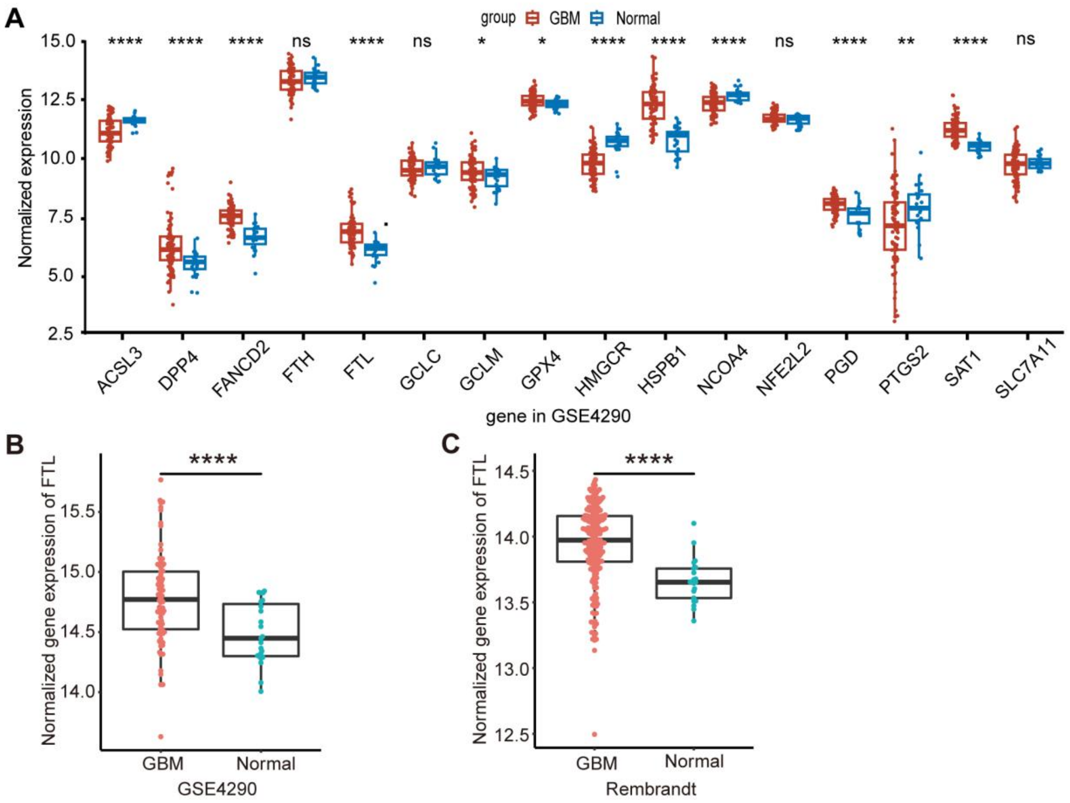

Figure S1. The expression of FTL in the GSE4290 and Rembrandt databases. (A) The expression level of ferroptosis-related genes was compared between normal and GBM tissue based on the GSE4290 database. **(B)** The expression of FTL was compared between normal and GBM tissue in the GSE4290 database. **(C)** The expression of FTL was compared between normal and GBM tissue in the Rembrandt database.

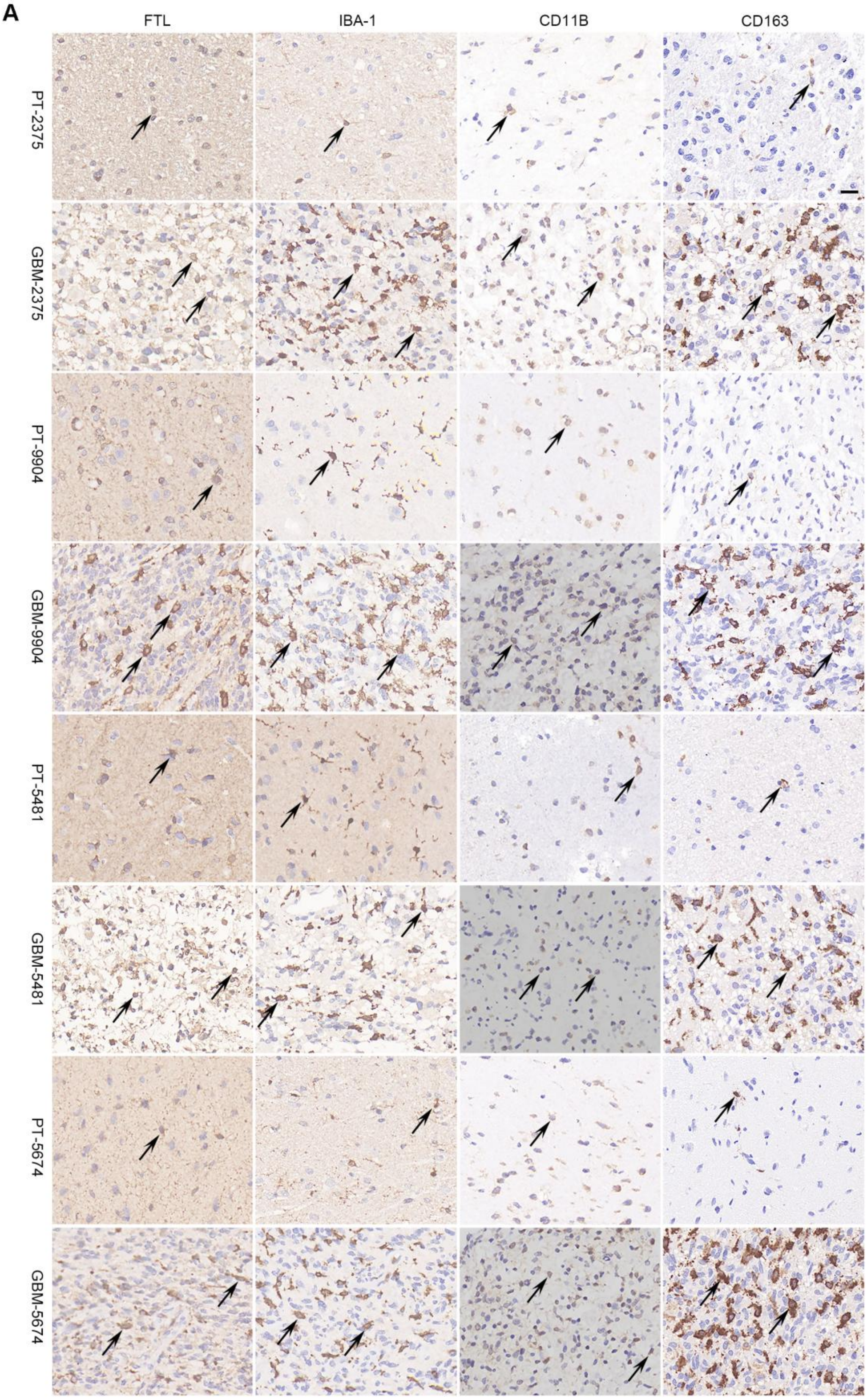

Figure S2. IHC images of protein expression levels in 4 GBM patients.

(A) IHC staining of FTL and microglial marker (IBA-1), macrophage

marker (CD11B) and M2 macrophage phenotype marker (CD163) in GBM-2375, GBM-9904, GBM-5481, GBM-5674 and corresponding PT (scale bar, 50 μm).

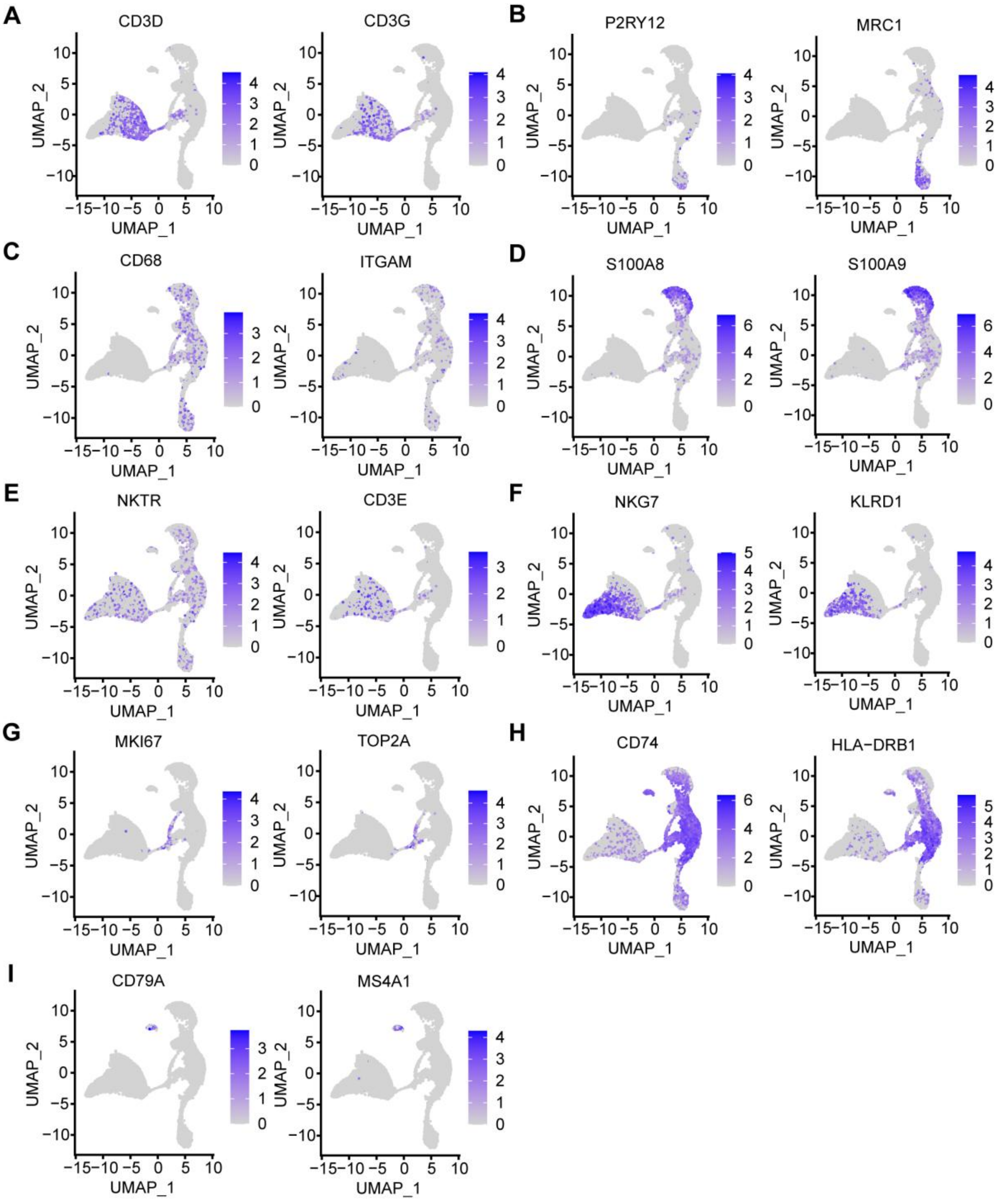

Figure S3. UMAPs of immune constituents of human glioma in GSE147275 database. UMAPs of immune constituents from patient glioma (n = 15) samples, showing clusters and representative markers for the following populations: **(A)** T cells (CD3D and CD3G), **(B)** Microglial (P2RY12 and MRC1), **(C)** TAM (CD68 and ITGAM), **(D)** Neutrophils (S100A8 and S100A9), **(E)** NKTs (NKTR, CD3E), **(F)** NK cells (KLRD1

and NKG7), **(G)** proliferative cells (MKI67, TOP2A), **(H)** APCs (CD74, HLA-DRB1) and **(I)** B cells (CD79A and MS4A7).

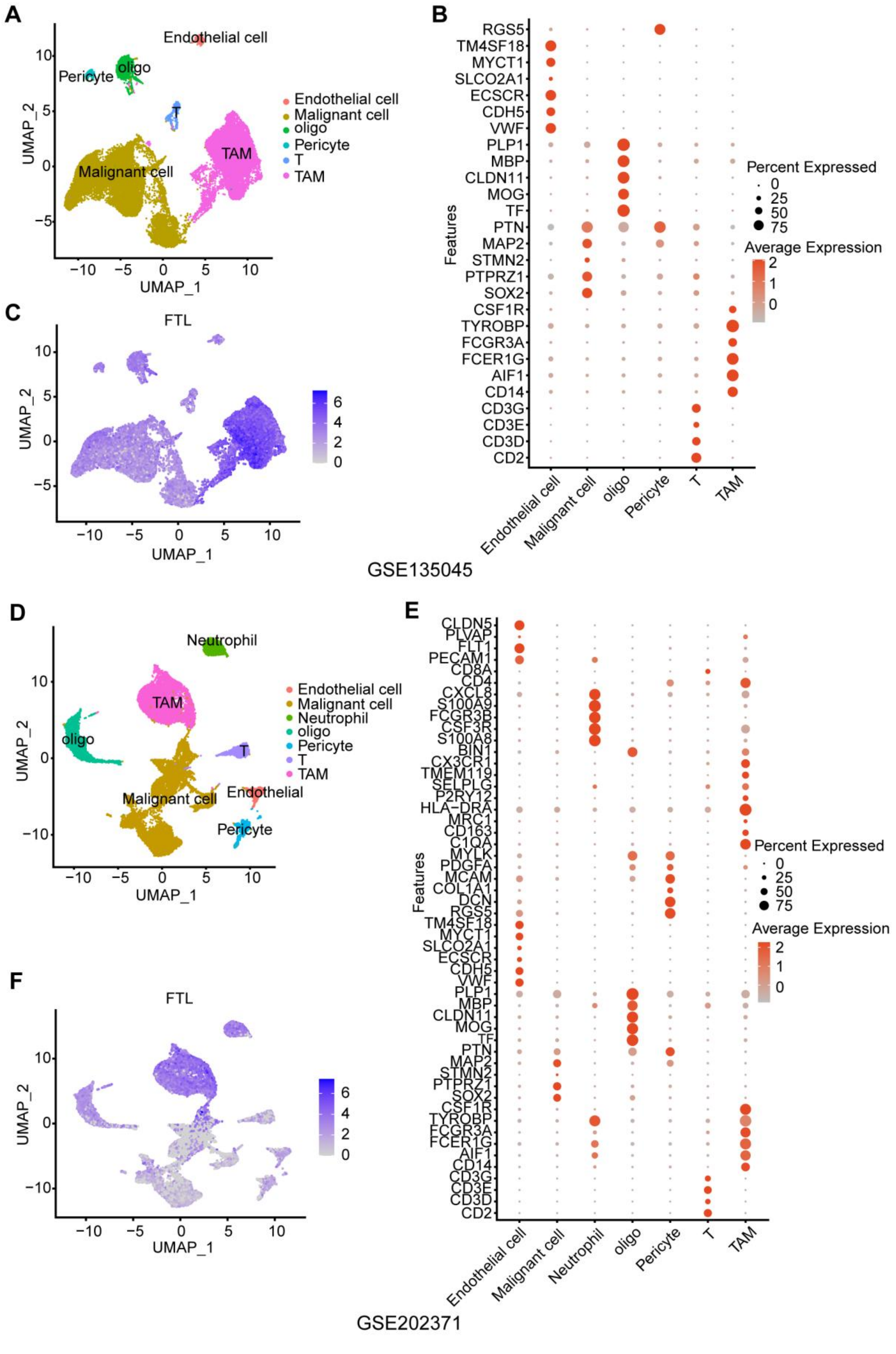

Figure S4. The enrichment of FTL in TAMs by single-cell analysis based on the GSE135045 and GSE202371 databases. (A) UMAPs of

immune constituents from glioma patients from GSE135045 database. **(B)** Representative markers for the immune constituents in the GSE135045 database. **(C)** The feature plot of FTL in UMAP in the GSE135045 database. **(D)** UMAPs of immune constituents from glioma patients from GSE202371 database. **(E)** Representative markers for the immune constituents in the GSE202371 database. **(F)** The feature plot of FTL in UMAP in the GSE202371 database.

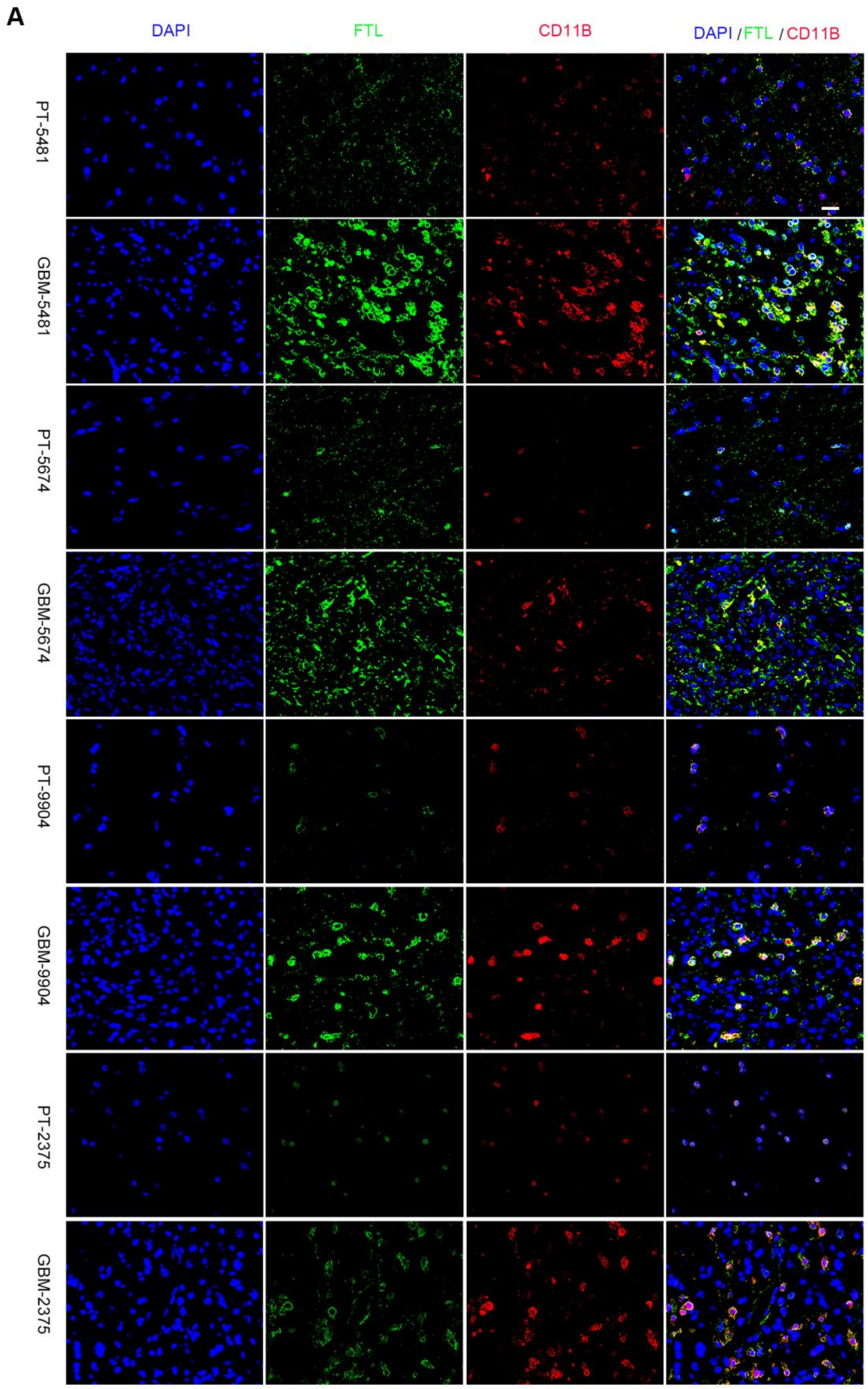

Figure S5. Immunofluorescence of protein expression levels in GBM patients. (A) Immunofluorescence staining of FTL and TAMs marker

(CD11B) in GBM-5481, GBM-5674, GBM-9904, GBM-2375 and corresponding PT (scale bar, 20 μm).

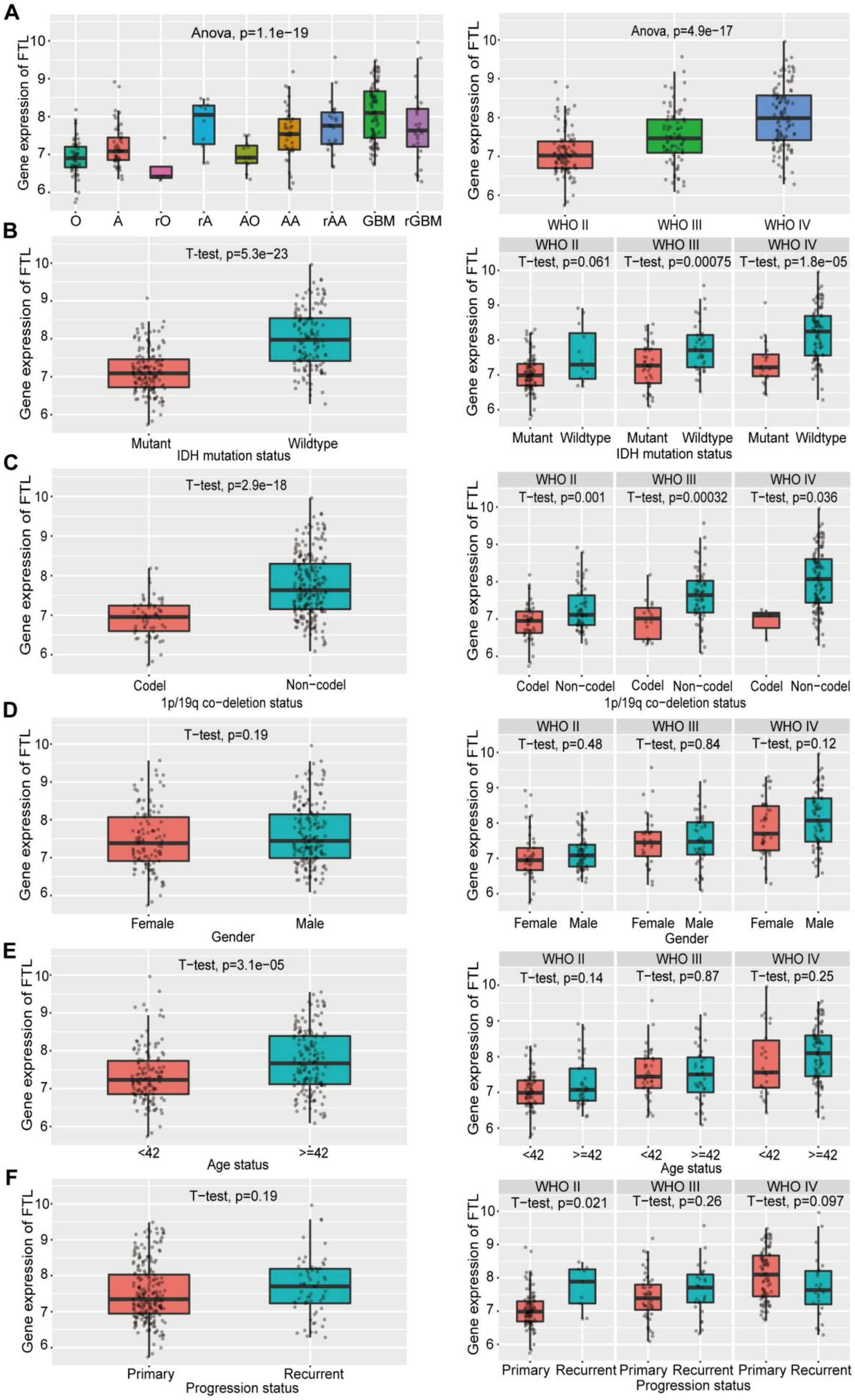

Figure S6. Validation of the clinical significance of FTL. The

correlation of FTL expression level with different types of gliomas and WHO grade **(A)**, IDH status **(B)**, 1p/19q codeletion status **(C)**, gender **(D)**, age **(E)** and progression status **(F)** in CGGA database.

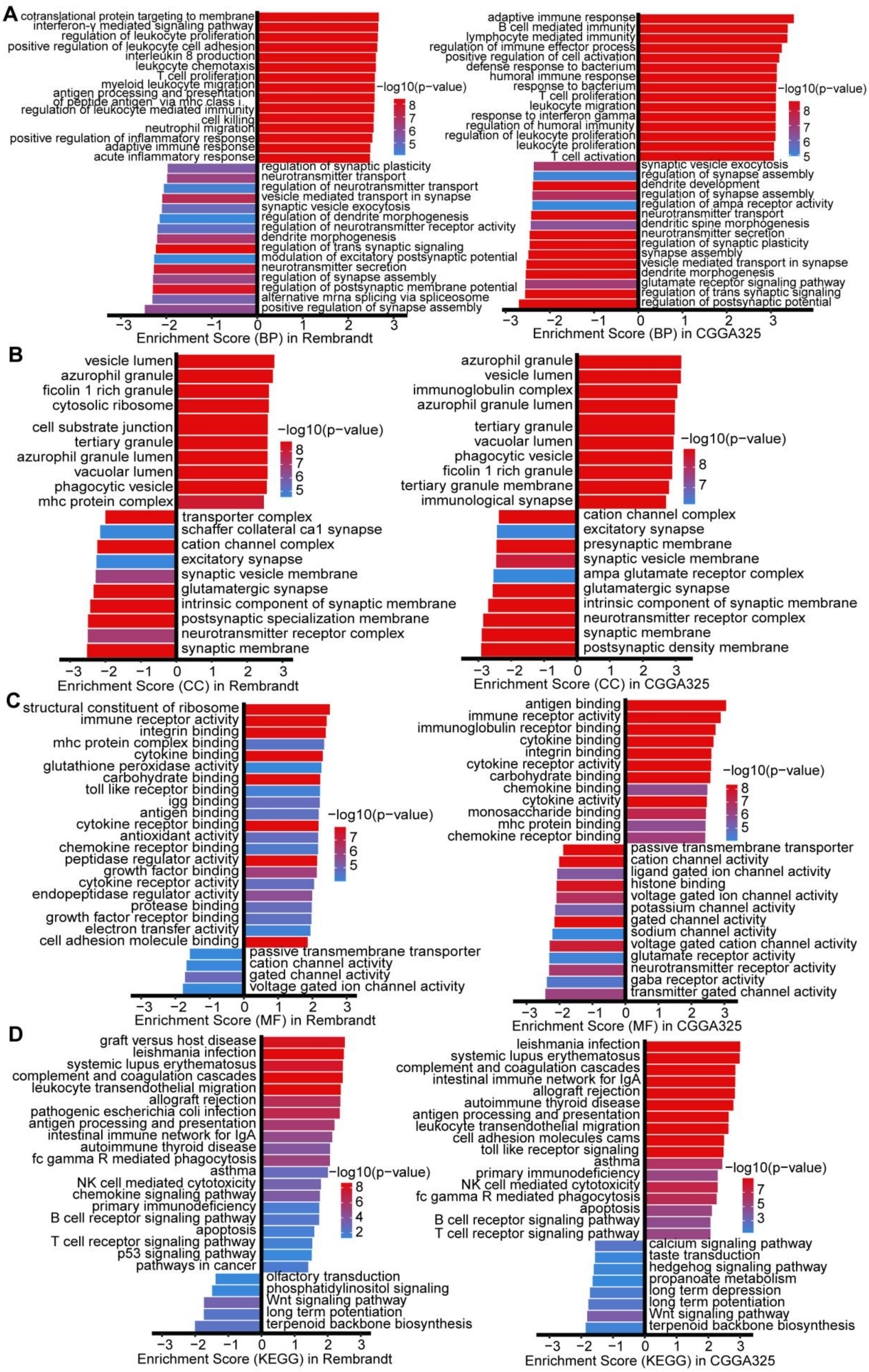

Figure S7. The co-expression network of FTL in glioma. (A-D) GO

terms for molecular function, cellular component, biological process, and KEGG pathways of FTL by GSEA analyses in GSE4290 and CGGA databases. Left panel shows GO and KEGG in the GSE4290 database. Right panel shows GO and KEGG in the CGGA database.

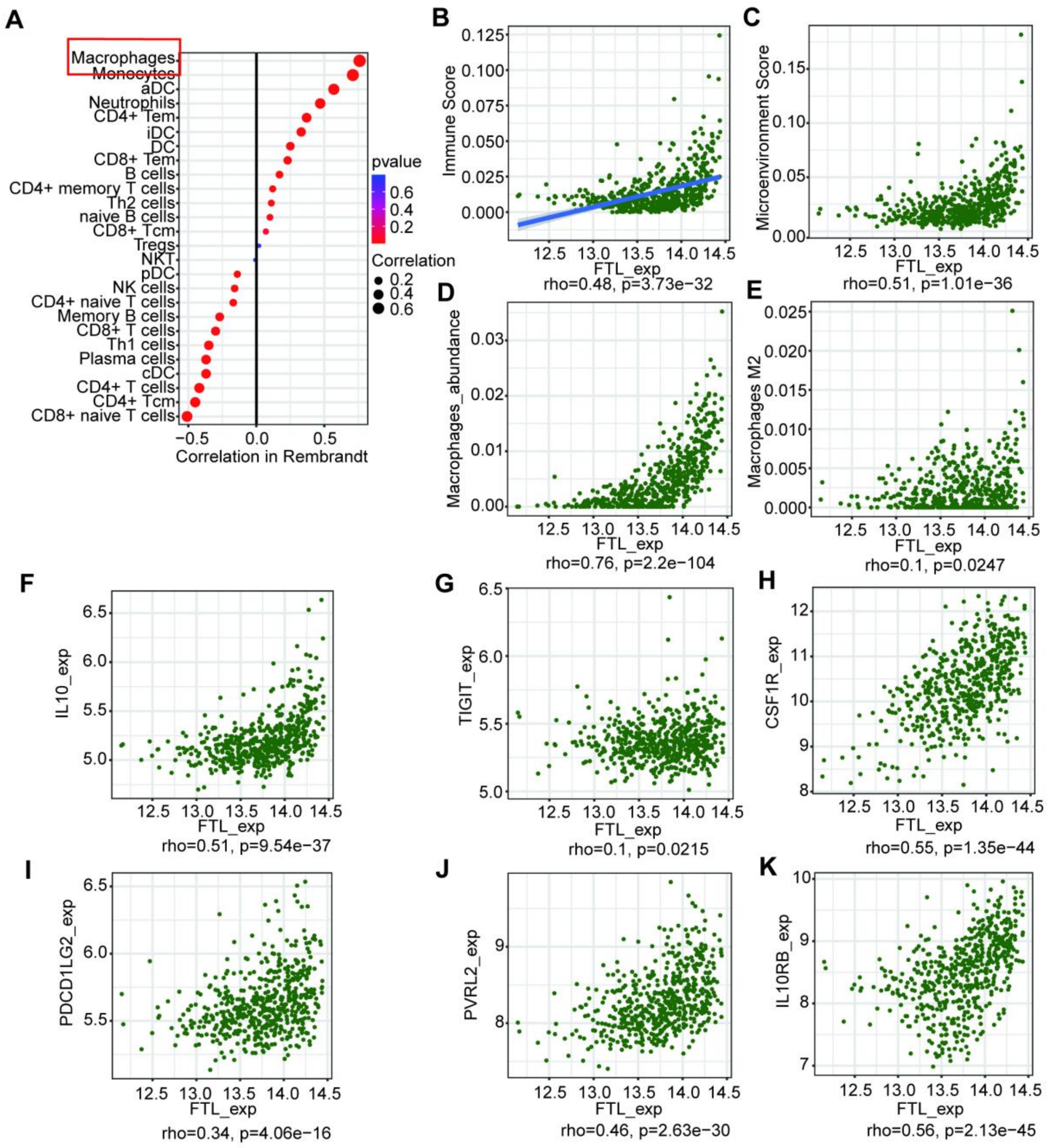

Figure S8. The role of FTL in the immune microenvironment of glioma based on the Rembrandt database. (A) Immune infiltration cells associated with FTL were performed using the ssGESA algorithm in the Rembrandt database. **(B-E)** Correlation analyses of FTL and immune score **(B)**, microenvironment score **(C)**, macrophages **(D)** and macrophages M2 **(E)** calculated by ESTIMATE algorithm were performed using Spearman's correlation test based on the Rembrandt

database. **(F-K)** Associations of the FTL expression with chemokines **(F)** and multiple immunoinhibitory molecules **(G-K)** from the Rembrandt database.

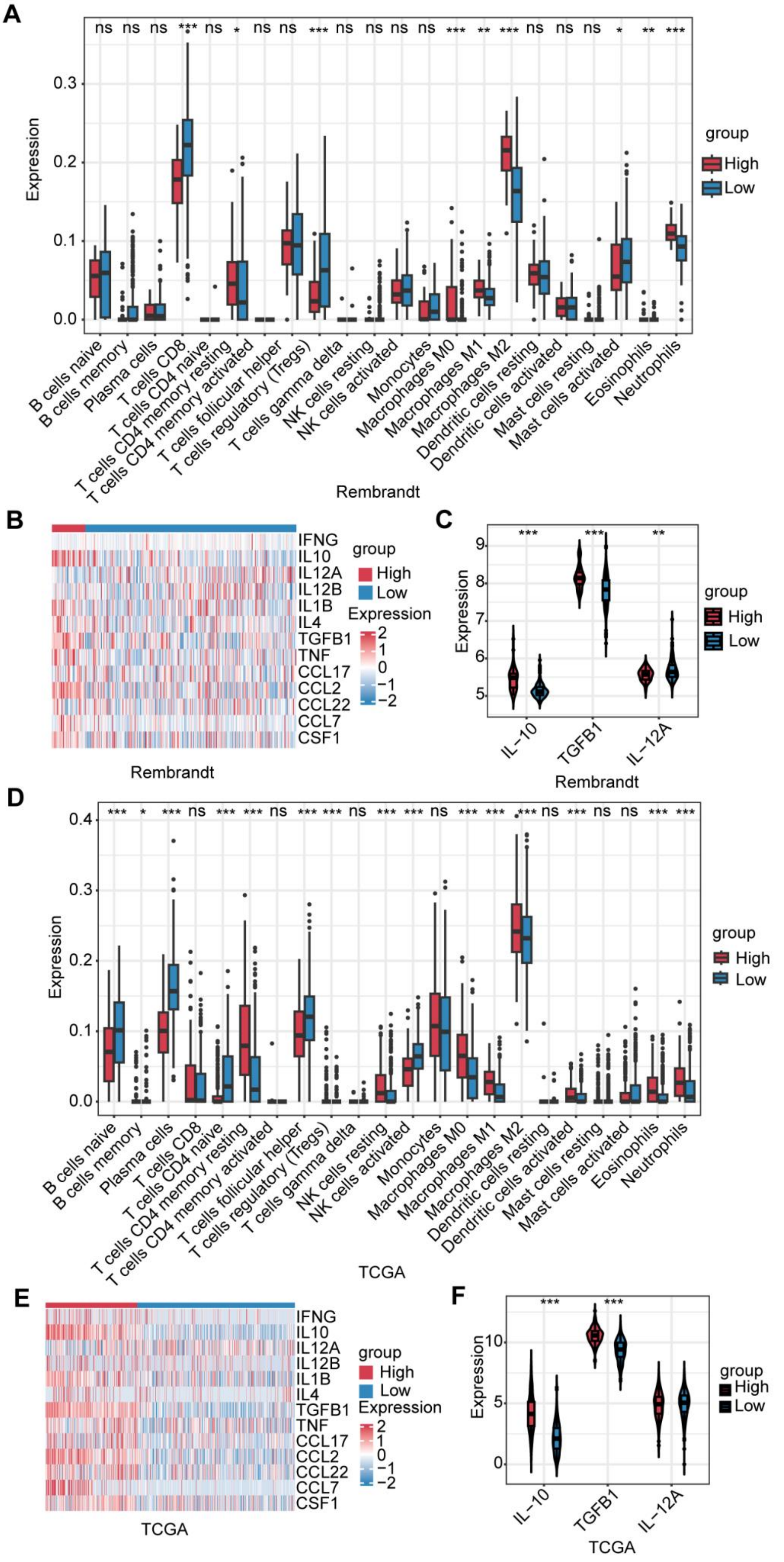
A
ns ns ns *** ns * ns ns *** ns ns ns ns *** ** *** ns ns ns * ** ***
0.3
0.2
0.1
0.0
Expression
group
High
Low
B cells naive
B cells memory
Plasma cells
T cells CD8
T cells CD4 naive
T cells CD4 memory resting
T cells CD4 memory activated
T cells follicular helper
T cells regulatory (Tregs)
T cells gamma delta
NK cells resting
NK cells activated
Monocytes
Macrophages M0
Macrophages M1
Macrophages M2
Dendritic cells resting
Dendritic cells activated
Mast cells resting
Mast cells activated
Eosinophils
Neutrophils
Rembrandt
B
IFNG
IL10
IL12A
IL12B
IL1B
IL4
TGFB1
TNF
CCL17
CCL2
CCL22
CCL7
CSF1
group
High
Low
Expression
2
1
0
−1
−2
Rembrandt
C
*** *** **
9
8
7
6
5
Expression
group
High
Low
IL−10
TGFB1
IL−12A
Rembrandt
D
*** * *** ns *** *** ns *** *** ns *** *** ns *** *** *** ns *** ns ns *** ***
0.4
0.3
0.2
0.1
0.0
Expression
group
High
Low
B cells naive
B cells memory
Plasma cells
T cells CD8
T cells CD4 naive
T cells CD4 memory resting
T cells CD4 memory activated
T cells follicular helper
T cells regulatory (Tregs)
T cells gamma delta
NK cells resting
NK cells activated
Monocytes
Macrophages M0
Macrophages M1
Macrophages M2
Dendritic cells resting
Dendritic cells activated
Mast cells resting
Mast cells activated
Eosinophils
Neutrophils
TCGA
E
IFNG
IL10
IL12A
IL12B
IL1B
IL4
TGFB1
TNF
CCL17
CCL2
CCL22
CCL7
CSF1
group
High
Low
Expression
2
1
0
−1
−2
TCGA
F
*** ***
10
5
0
Expression
group
High
Low
IL−10
TGFB1
IL−12A
TCGA

Figure S9. Bioinformatics analysis of the role of shFTL on major immune cell populations in glioma microenvironment based on the Rembrandt and TCGA-glioma databases. (A) Scores of immune cells were compared between high-FTL and low-FTL groups in the Rembrandt database. **(B)** The heatmap showed that cytokines levels were compared between high-FTL and low-FTL groups in the Rembrandt database. **(C)** The expressions of IL-10, TGFB1 and IL-12A were compared between high-FTL and low-FTL groups in the Rembrandt database. **(D)** Scores of immune cells were compared between high-FTL and low-FTL groups in the TCGA-glioma database. **(E)** The heatmap showed that gene expressions of cytokines levels were compared between high-FTL and low-FTL groups in the TCGA-glioma database. **(F)** The gene expressions of IL-10, TGFB1 and IL-12A were compared between high-FTL and low-FTL groups in the TCGA-glioma database.

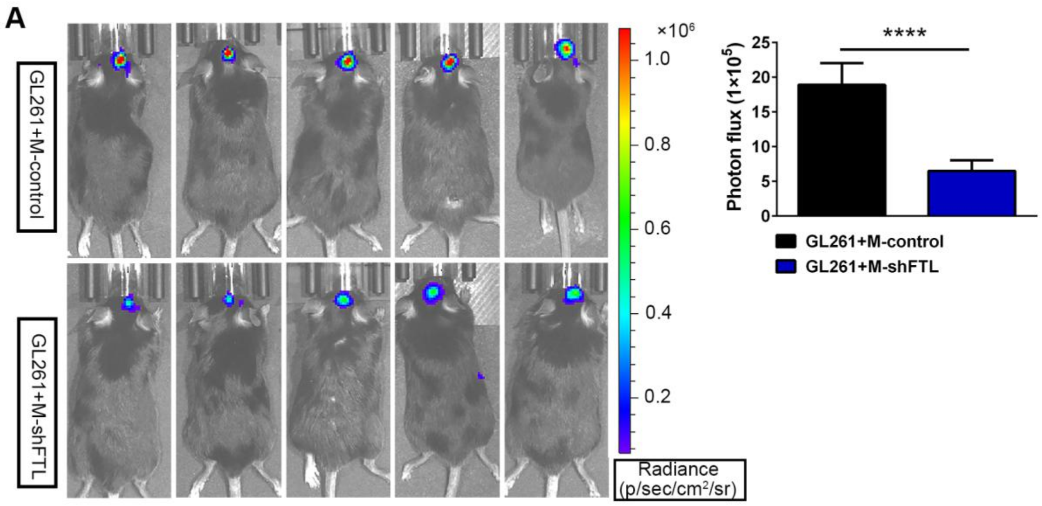

Figure S10. The role of shFTL in tumor growth by bioluminescent imaging. (A) In vivo bioluminescent imaging analysis of tumor growth in xenograft C57BL/6N mice bearing GL261 glioma cells with M-control and M-shFTL. Representative images on day 21 post-implantation are shown, and quantification was performed.

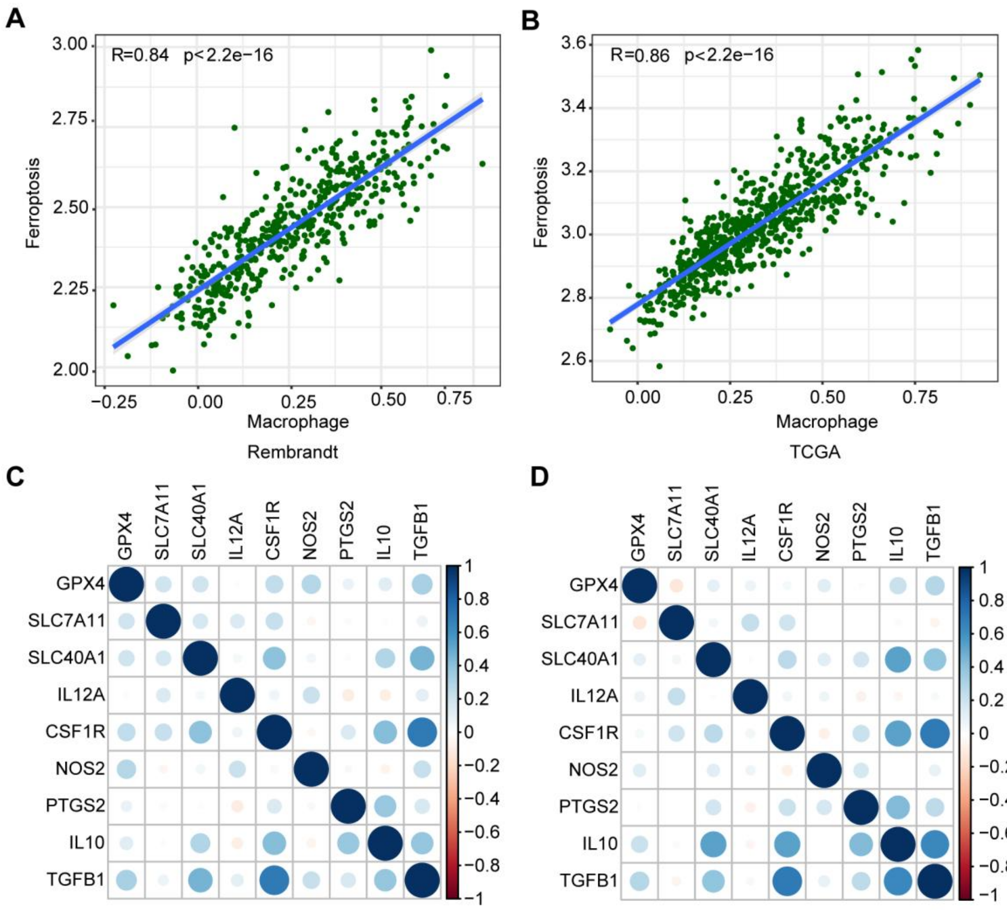

Figure S11. The correlation analysis between ferroptosis and macrophage polarization based on Rembrandt and TCGA-glioma databases. (A) Ferroptosis was significantly correlated with macrophage polarization (R = 0.84) in Rembrandt database. **(B)** Ferroptosis was significantly correlated with macrophage polarization (R = 0.86) in TCGA-glioma database. **(C)** The correlation analysis between ferroptosis-related genes (GPX4, SLC7A11 and SLC40A1) and macrophage markers M1 (IL12A, NOS2, PTGS2) and M2 (IL10, TGFB1, CSF1R) in Rembrandt database. **(D)** The correlation analysis between ferroptosis-related genes and macrophage markers M1 and M2 in

TCGA-glioma database.

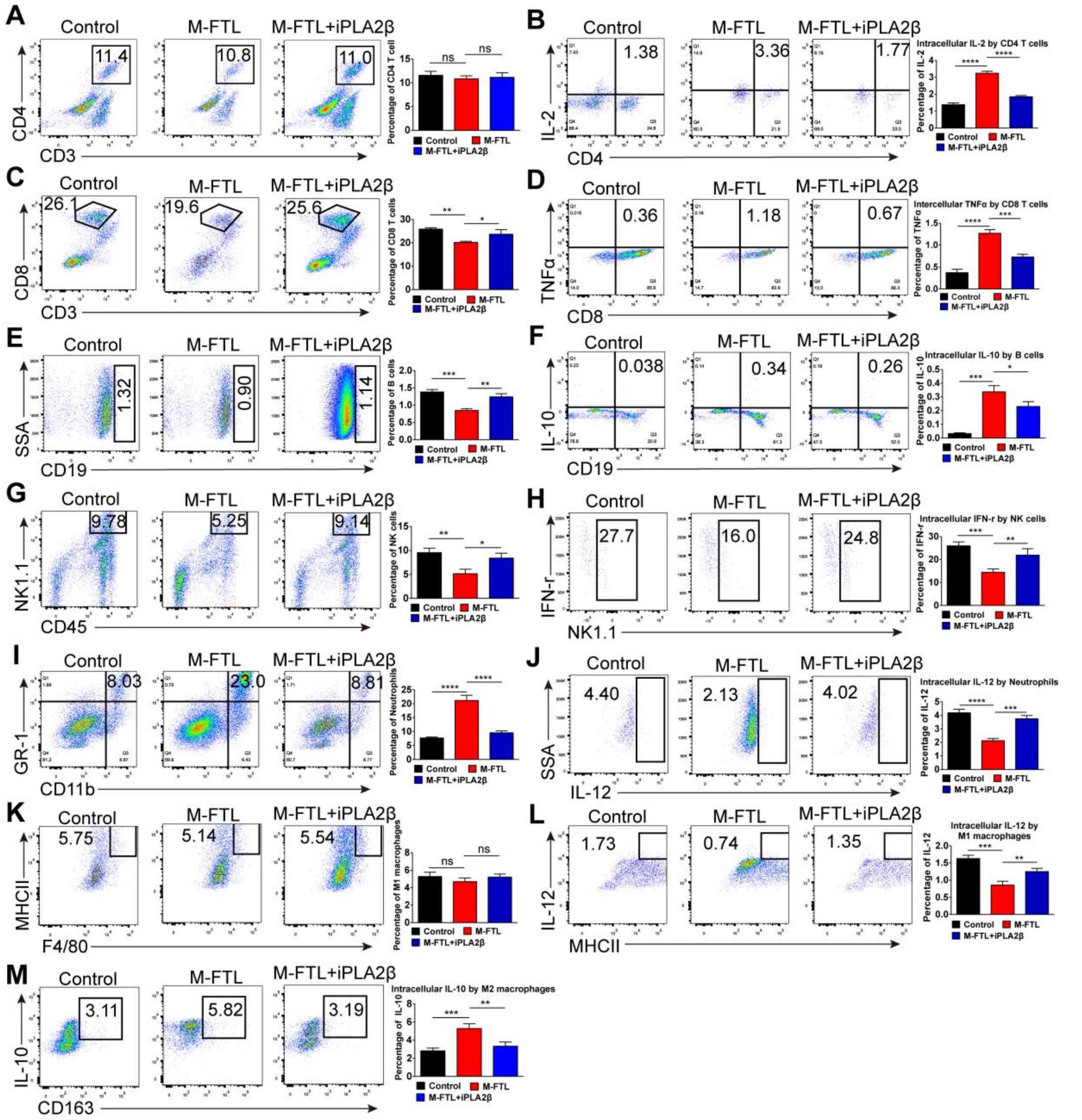

Figure S12. The role of shFTL on major immune cell populations in glioma microenvironment was assessed by flow cytometry analysis. **(A)** The proportions of CD4$^+$ T cells from xenograft mouse brains with GL261 and M-control, M-FTL or M-FTL+iPLA2β were determined by flow cytometry. **(B)** The proportions of IL-12$^+$ CD4 T cells from xenograft mouse brains with GL261 and M-control, M-FTL or M-FTL+iPLA2β were compared. **(C)** The proportions of CD8$^+$ T cells from xenograft mouse brains with GL261 and M-control, M-FTL or

M-FTL+iPLA2β were determined by flow cytometry. **(D)** The proportions of TNFα$^+$ CD8 T cells from xenograft mouse brains with GL261 and M-control, M-FTL or M-FTL+iPLA2β were compared. **(E)** The proportions of B cells from xenograft mouse brains with GL261 and M-control, M-FTL or M-FTL+iPLA2β were determined by flow cytometry. **(F)** The proportions of IL-10$^+$ B cells from xenograft mouse brains with GL261 and M-control, M-FTL or M-FTL+iPLA2β were compared. **(G)** The proportions of NK cells from xenograft mouse brains with GL261 and M-control, M-FTL or M-FTL+iPLA2β were determined by flow cytometry. **(H)** The proportions of IFN-γ$^+$ NK cells from xenograft mouse brains with GL261 and M-control, M-FTL or M-FTL+iPLA2β were compared. **(I)** The proportions of neutrophils from xenograft mouse brains with GL261 and M-control, M-FTL or M-FTL+iPLA2β were determined by flow cytometry. **(J)** The proportions of IL-12$^+$ neutrophils from xenograft mouse brains with GL261 and M-control, M-FTL or M-FTL+iPLA2β were compared. **(K)** The proportions of M1 macrophages from xenograft mouse brains with GL261 and M-control, M-FTL or M-FTL+iPLA2β were determined by flow cytometry. **(L)** The proportions of IL-12$^+$ M1 macrophages from xenograft mouse brains with GL261 and M-control, M-FTL or M-FTL+iPLA2β were compared. **(M)** The proportions of IL-10$^+$ M2 macrophages from xenograft mouse brains with GL261 and M-control,

M-FTL or M-FTL+iPLA2β were compared.

Table S1. Primers used for qPCR analysis.

Gene name	Primer orientation	Sequences	Amplicon size
CD163 (human)	Forward	TGAAGTGAAGTGCAAAGGGAATG	192
	Reverse	CCAAGGATCCCGACTGCAATAAA	
IL-1β (human)	Forward	AGCACCTTCTTTCCCTTCATCTT	201
	Reverse	CACCACTTGTTGCTCCATATCCT	
IL-10 (human)	Forward	AGCTCCAAGAGAAAGGCATCTAC	121
	Reverse	GTCTATAGAGTCGCCACCCTGAT	
INOS (human)	Forward	CCAAGCTCTACACCTCCAATGT	164
	Reverse	GCTGGATGTCGGACTTTGTAGAT	
STAT6 (human)	Forward	GCTGGATGTCGGACTTTGTAGAT	248
	Reverse	GTGGCTTTGGCATTGTTGTCT	
TNFα (human)	Forward	CCATGTTGTAGCAAACCCTCAAG	150
	Reverse	AAGAGGACCTGGGAGTAGATGAG	
CD163 (mouse)	Forward	AAGAGGACCTGGGAGTAGATGAG	126
	Reverse	AAGAGGACCTGGGAGTAGATGAG	
IL-1β (mouse)	Forward	AAGAGGACCTGGGAGTAGATGAG	150
	Reverse	AAGAGGACCTGGGAGTAGATGAG	
IL-10 (mouse)	Forward	AAGAGGACCTGGGAGTAGATGAG	129
	Reverse	AAGAGGACCTGGGAGTAGATGAG	
INOS (mouse)	Forward	AAGAGGACCTGGGAGTAGATGAG	182
	Reverse	AAGAGGACCTGGGAGTAGATGAG	
STAT6 (mouse)	Forward	AAGAGGACCTGGGAGTAGATGAG	144
	Reverse	AAGAGGACCTGGGAGTAGATGAG	
TNFα (mouse)	Forward	AAGAGGACCTGGGAGTAGATGAG	223
	Reverse	AAGAGGACCTGGGAGTAGATGAG	
